# Increase of secondary metabolites in sweet basil (*Ocimum basilicum* L.) leaves by exposure to N_2_O_5_ with plasma technology

**DOI:** 10.1038/s41598-024-63508-8

**Published:** 2024-06-04

**Authors:** Rie Tateishi, Natsumi Ogawa-Kishida, Nobuharu Fujii, Yuji Nagata, Yoshiyuki Ohtsubo, Shota Sasaki, Keisuke Takashima, Toshiro Kaneko, Atsushi Higashitani

**Affiliations:** 1 Graduate School of Life Sciences, Tohoku University, Sendai, 980-8577 Japan; 2 Graduate School of Engineering, Tohoku University, Sendai, 980-8579 Japan

**Keywords:** Dinitrogen pentoxide, Metabolomics, Plasma technology, Secondary metabolites, Transcriptional upregulation, Plasma physics, Plant stress responses, Secondary metabolism

## Abstract

Exposure to N_2_O_5_ generated by plasma technology activates immunity in Arabidopsis through tryptophan metabolites. However, little is known about the effects of N_2_O_5_ exposure on other plant species. Sweet basil synthesizes many valuable secondary metabolites in its leaves. Therefore, metabolomic analyses were performed at three different exposure levels [9.7 (Ex1), 19.4 (Ex2) and 29.1 (Ex3) μmol] to assess the effects of N_2_O_5_ on basil leaves. As a result, cinnamaldehyde and phenolic acids increased with increasing doses. Certain flavonoids, columbianetin, and caryophyllene oxide increased with lower Ex1 exposure, cineole and methyl eugenol increased with moderate Ex2 exposure and l-glutathione GSH also increased with higher Ex3 exposure. Furthermore, gene expression analysis by quantitative RT-PCR showed that certain genes involved in the syntheses of secondary metabolites and jasmonic acid were significantly up-regulated early after N_2_O_5_ exposure. These results suggest that N_2_O_5_ exposure increases several valuable secondary metabolites in sweet basil leaves via plant defense responses in a controllable system.

## Introduction

We have recently developed a portable plasma device capable of selectively synthesizing high concentrations of dinitrogen pentoxide (N_2_O_5_) only from the atmosphere by atmospheric pressure plasma (APP) technology^1^. Nitrogen oxide (NOx) is mainly produced in high-temperature plasma reactor with high gas temperature (> 1000 K), while ozone (O_3_) is produced in low-temperature plasma reactor with low gas temperature (< 400 K). By optimizing the mixing conditions of these gases, it is possible to selectively synthesize highly concentrated N_2_O_5_. N_2_O_5_ gas quickly reacts with water to generate reactive intermediate species (*e.g.*, NO_2_^+^_aq_, [NO_2_^+^·NO_3_^−^]_aq_), finally converted into NO_3_^−^ ions^2^, which can be used as a nitrogen source fertilizer for plants^3^. In other words, N_2_O_5_ does not have the residual properties found in other chemicals. Furthermore, exposure to N_2_O_5_ gas activates plant immunity and suppresses both *Botrytis cinerea* infection and propagation of Cucumber mosaic virus strain yellow in Arabidopsis^4^. Besides, several genes involved in jasmonic acid (JA) and ethylene signaling pathways and the synthesis of secondary metabolites from tryptophan metabolism are immediately activated by N_2_O_5_ exposure in Arabidopsis^4,5^. However, whether exposure to N_2_O_5_ gas affects the biosynthesis of secondary metabolites in plants other than Arabidopsis requires further investigation.

Secondary metabolites are multifunctional organic compounds that play important and defensive roles in plant stress adaptability and resilience. Some of these compounds may be involved in the regulation of several immune responses that are evolutionarily conserved in the plant kingdom, such as callose deposition and programmed cell death^6,7^. JA and ethylene present an interesting example of synergism and antagonism, acting as multiple signaling networks regulating stress responses involving secondary metabolites^7^. Several plant species, such as the Lamiacease, Asteracease, and Solanaceae, have glandular trichomes in which they accumulate essential oils containing several secondary metabolites such as terpenoids and phenylpropenes^8–10^. Essential oils play an important role in protecting plants from insects, bacteria, and fungi. Moreover, these metabolites also contribute significantly to human health and medicine as nutraceuticals, pharmaceuticals and supplements^11–14^. Here, we assessed whether exposure to N_2_O_5_ alters the biosynthesis of secondary metabolites such as flavonoids, terpenoids and phenylpropenes in sweet basil (*Ocimum basilicum* L.) leaves. Metabolomic analyses were performed using non-targeted Liquid Chromatography Mass Spectrometry (LC–MS) and targeted Gas Chromatography Mass Spectrometry (GC–MS). In addition, the expression levels of enzyme genes involved in the biosynthesis of these metabolites were examined.

## Results

### Effects of N_2_O_5_ exposure on plant growth and biosynthesis of secondary metabolites

**Figure 1:**
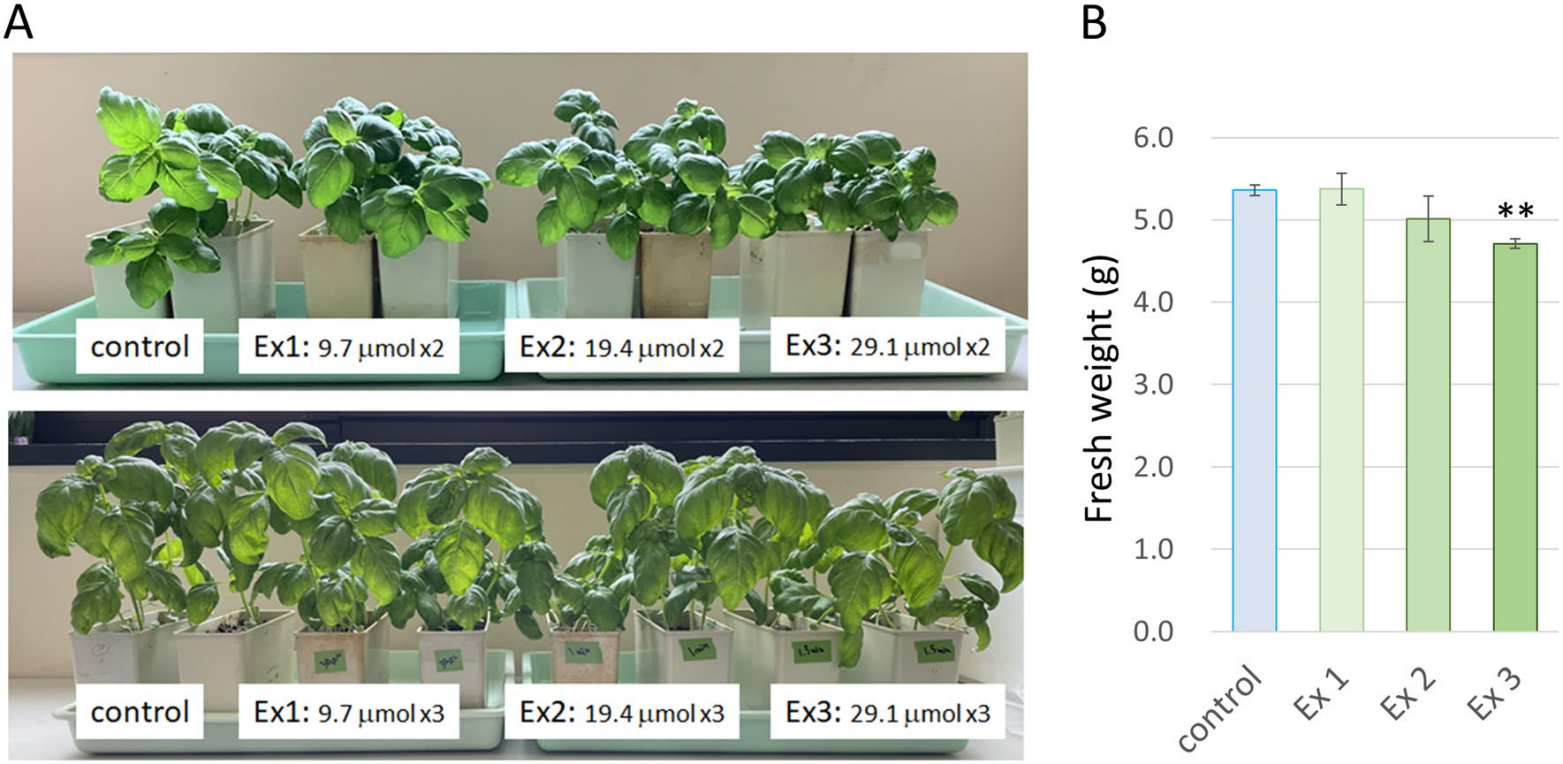
Effect of N_2_O_5_ exposure on growth of sweet basil. (**A**) Basil plants before the third N_2_O_5_ exposure (upper panel: 4-weeks old after sowing) and 8 days after exposure (lower panel). (**B**) Shoot fresh weight of approximately 32-day-old plants, 8 days after the third exposure. Error bars represent SE. ** indicates a significant difference at *p* < 0.01 by Student's *t*-test*.*

N_2_O_5_ inhibited growth and caused a decrease in basil plant height and fresh weight depending on the exposure level (Fig. 1A, B). At the highest exposure level, Ex3 (29.1 μmol per exposure: 0.32 μmol/s for 90 s), fresh weight was reduced by about 10% in plants 8 days after the third exposure (plants about 32 days old). To assess the effect of N_2_O_5_ on the biosynthesis of secondary metabolites, non-targeted metabolomic analysis of these basil leaf hydrophilic metabolites, focusing mainly on phenylpropanoids and flavonoids, was performed using LC–MS-based MS-derived data (mass spectra). A total of 3,530 MS peaks were identified from the analysis of three independent plants under different N_2_O_5_ exposure conditions, in addition to the non-exposed control group. Of these, 151 compounds were annotated at the molecular level, of which 23 compounds significantly increased in content in basil leaves under all N_2_O_5_ treatment conditions, while 15 compounds decreased (Supplementary Table 1). Among others, l-glutathione GSH content showed a significant tendency to increase in dependence on N_2_O_5_ exposure (Supplementary Table 1).

**Figure 2:**
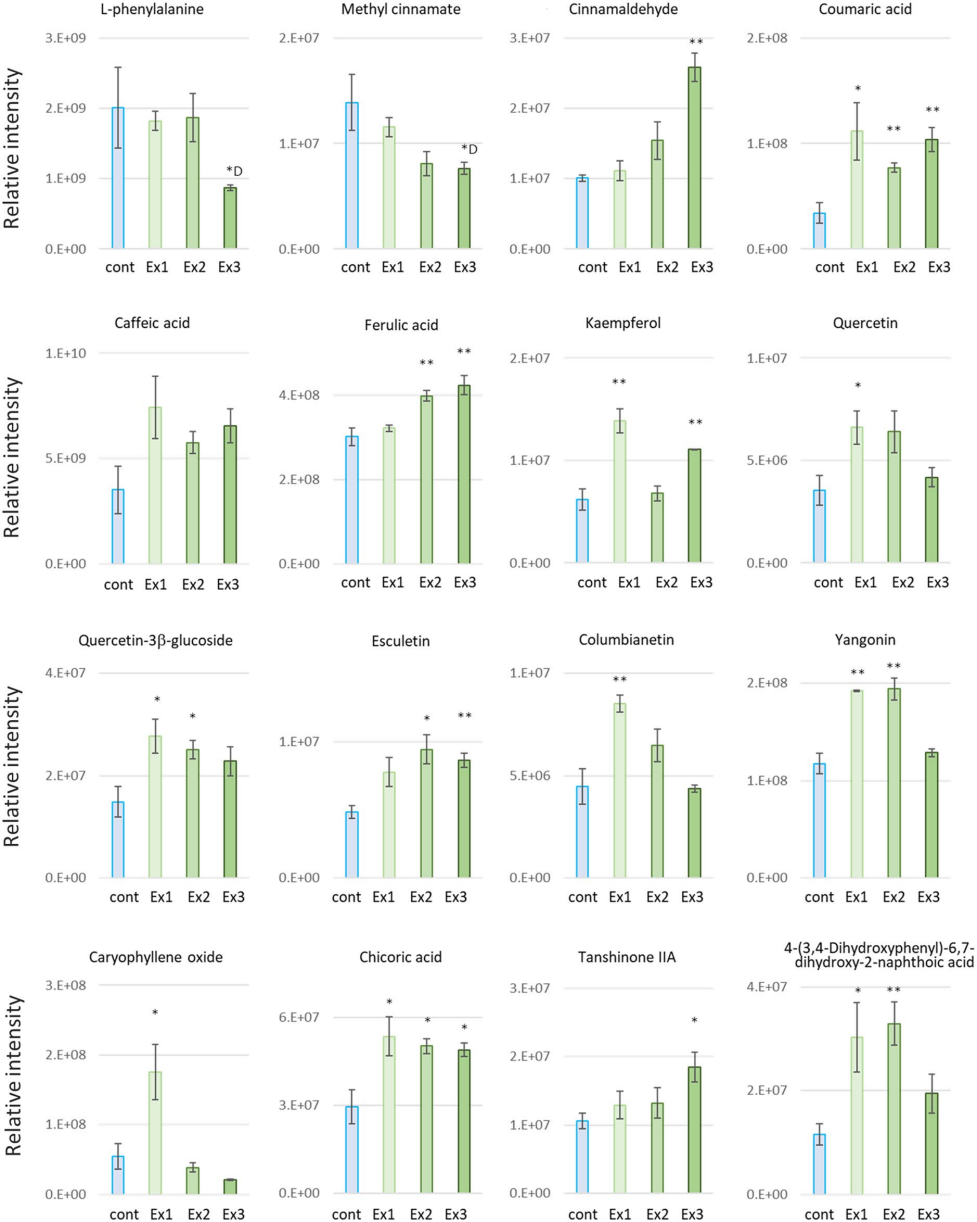
Metabolome analysis using non-targeted LC–MS analysis. Certain phenylpropenes altered by N_2_O_5_ exposure in 32-day-old plant leaves. In particular, coumaric acid, caffeic acid, Kaempferol and Quercetin-3β-glucoside significantly increased in at least two different exposure conditions. AMP and Kynurenic acid were not changed. Data represent at least three biological samples in each metabolite and are mean ± S.E. Student’s *t*-test compared with each control level (*p < 0.05, **p < 0.01, D: downregulated).

Interestingly, among the compounds that showed significant changes, some phenolic acids, e.g. coumaric acid, caffeic acid and ferulic acid, which are metabolic intermediates of some plant secondary metabolites, tended to increase with increasing N_2_O_5_ exposure compared to controls (Fig. 2). In contrast, l-phenylalanine, the substrate of these phenolic acids, tended to decrease significantly under Ex3 conditions and slightly under other conditions when exposed to N_2_O_5_. Furthermore, methyl cinnamate tended to decrease in a dose-dependent manner with N_2_O_5_ exposure, whereas cinnamaldehyde, the main compound in cinnamon essential oil and having excellent antibacterial activity, tended to increase in opposite direction (Fig. 2). Tanshinone IIA derived from phenanthrene-quinone was significantly increased by Ex3 exposure. Certain flavonoids, kaempferol, quercetin and quercetin-3β-glucoside, increased with low Ex1 exposure (9.7 μmol per exposure: at 0.32 μmol/s for 30 s) and conversely showed a tendency to decrease with further increases in N_2_O_5_ dose. One volatile essential oil component, an oxygenated terpenoid, caryophyllene oxide increased with low Ex1 exposure and conversely showed a tendency to decrease with further increases in N_2_O_5_ dose. Certain flavonoids, kaempferol, quercetin and quercetin-3β-glucoside, and columbianetin also showed a similar trend of increasing with lower Ex1 exposure and decreasing with increasing exposure (Fig. 2). An oleralignan 4-(3,4-dihydroxyphenyl)-6,7-dihydroxy-2-naphthoic acid, a type of lignans, and yangonin increased significantly with Ex1 and Ex2 (19.4 μmol per exposure: at 0.32 μmol/s for 60 s) exposure. Esculetin and chicoric acid increased under almost all exposure conditions.

**Figure 3:**
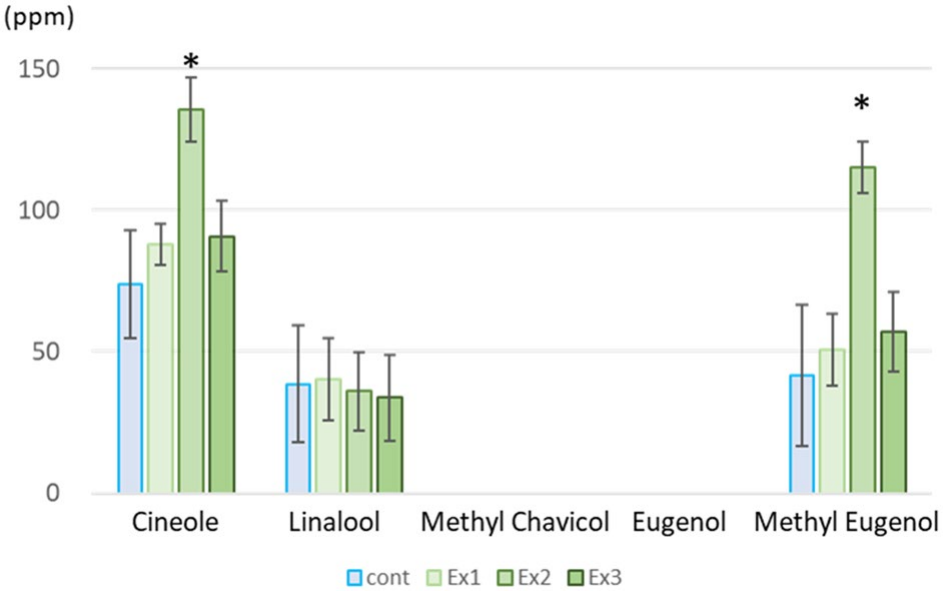
Targeted-GC–MS analysis for volatile organic compounds. The contents of cineole, linalool, methyl chavicol, eugenol, and methyl eugenol (ppm / fresh leaves) were determined by GC–MS using standard solutions of each compound. Data represent at least three biological samples of 32-day-old plant leaves for each metabolite and are means ± S.E. Student’s *t-*test compared with each control level (*p < 0.05).

Five representative essential oil constituents, cineole, linalool, methyl chavicol, eugenol and methyl eugenol, which were not identified in the non-targeted metabolomic analysis, were then examined by targeted GC–MS analysis of basil leaf extracts. The results showed that cineol and methyl eugenol were significantly increased under moderate Ex2 condition (Fig. 3). These compounds also showed higher mean values in the Ex1 and Ex3 conditions compared to the control, but the inter-individual variability in the same experimental conditions was also large and not significant. Conversely, linalool showed a slight but not significant decreasing trend with N_2_O_5_ exposure. Under these experimental conditions, eugenol concentrations were below the detection limit (Fig. 3). Note that this sweet basil strain is a methyl eugenol producing strain and methyl chavicol was not detected. These metabolomic analyses were set up as biological three-sample analyses for each exposure condition, and although increasing and decreasing trends were observed, some showed not significant different compared to non-irradiated controls. Only those showing significant differences compared to non-exposed controls are marked with an asterisk (Fig. 2, 3, Supplementary Table 1).

## Effects of N_2_O_5_ exposure on secondary metabolite biosynthesis gene expression

Eight days after the third exposure, RNA was extracted from these approximately 32-day-old plants and analyzed by RT-PCR for changes in gene expression associated with the biosynthesis of secondary metabolites, particularly phenylpropanoids. The genome of sweet basil is tetraploid, not all genes have been fully decoded and the number of paralogous and pseudogenes in the genome is unknown^15^. Therefore, the gene expression status of the basil leaves used in this study under normal conditions was investigated by RNA sequencing, and several sequences of genes involved in the biosynthesis of secondary metabolites with relatively high expression levels and significant homology were identified (Supplementary Table 2). According to the determined sequences, respective primer sets were synthesized for their quantitative RT-PCR expression analysis (Supplementary Table 2). According to the determined sequences, respective primer sets were synthesized in highly conserved regions for quantitative RT-PCR expression analysis of several paralogues at once. In addition, previously published basil genes^16–18^, cinnamate 4-hydroxilase (*C4H*), 4-coumaric acid coenzyme A ligase (*4CL*), cinnamyl alcohol dehydrogenase (*CAD*), coniferyl alcohol acetyl transferase (*CAAT*) and eugenol synthases (*EGS*) primer sets could be used for this experiment.

**Figure 4:**
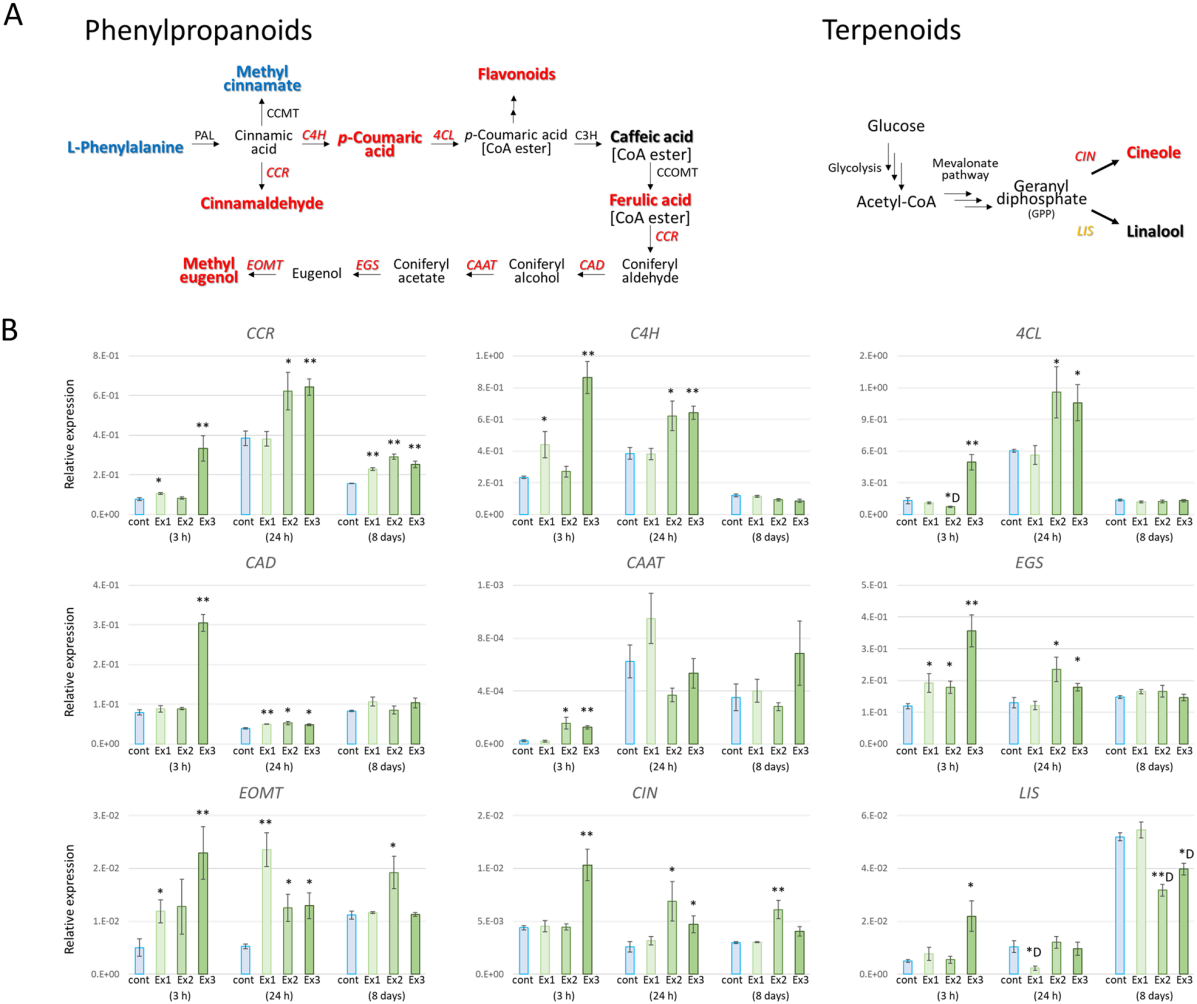
Expression levels of secondary metabolite biosynthesis genes in basil exposed to N_2_O_5_ gas. (**A**) Phenylpropanoids, cinnamaldehyde and methyl eugenol biosynthesis pathway and enzymes: abbreviations: PAL, phenylalanine ammonia lyase; CCMT, *p*-coumarate/cinnamate carboxyl methyltransferase; CCR, cinnamoyl-CoA reductase; C4H, cinnamate 4-hydroxylase; 4CL, 4-coumarate CoA ligase; C3H, *p*-coumarate 3-hydroxylase; CCOMT, caffeoyl-CoA *O*-methyltransferase; CAD, cinnamyl alcohol dehydrogenase; CAAT, coniferyl alcohol acetyl transferase; EGS, eugenol (and chavicol) synthase; EOMT, eugenol *O*-methyltransferase. Terpenoids, cineole and linalool biosynthesis pathway and enzymes: abbreviation: CIN, 1,8-cineole synthase; LIS: *S*-linalool synthase. (**B**) Real-time quantitative RT-PCR analysis of *CCR, C4H, 4CL, CAD, CAAT, EGS, EOMT, CIN* and *LIS* gene was performed in 14-day-old plants 3 and 24 h after the first exposure to N_2_O_5_ and in 32-day-old plants 8 days after the third exposure to N_2_O_5_ using gene-specific primers and relative expression ratios were calculated using *β-tubulin* gene expression (Supplementary Table 2). Data represent at least three biological samples with three technical replicates in each gene expression and are mean ± S.E. Student’s *t*-test compared with each control level (*p < 0.05, **p < 0.01, D: downregulated). Red color: upregulated; Orange: up and down regulated; Blue: downregulated.

Interestingly, the expression of the cinnamoyl-CoA reductase (*CCR*) gene was significantly increased by N_2_O_5_ exposure in approximately 32-day-old plant leaves, even under Ex1 low-exposure conditions, reaching a plateau at Ex2 exposure (Fig. 4). *CIN* and eugenol *O*-methyltransferase (*EOMT*) were also significantly elevated under the same Ex2 conditions, but not under other conditions (Fig. 4). On the other hand, no other changes in gene expression involved in the biosynthetic process of a series of secondary metabolites were observed (Fig. 4). It was suggested that this may be due to the relatively rapid response of gene expression fluctuations, which may have returned to a steady state 8 days after the last exposure, except for some genes such as *CCR*. Therefore, gene expression was also analyzed for approximately 14-day-old plants (when secondary set of leaves began to emerge), 3 and 24 h after the first exposure to N_2_O_5_ gas. As a result, a significant increase in the expression of biosynthetic genes for secondary metabolites of *CCR*, *C4H*, *4CL*, *CAD*, *CAAT*, *EGS*, *EOMT*, *CIN* and *S*-linalool synthase (*LIS*), all of which could be investigated in this study, was observed 3 h after exposure, which showed an exposure dose-dependent trend (Fig. 4). Interestingly, some genes showed increased expression after 24 h, while some showed a decrease, suggesting that the induction at many gene expression levels is a transient and variable response following N_2_O_5_ exposure.

## JA signaling in N_2_O_5_ exposure

**Figure 5:**
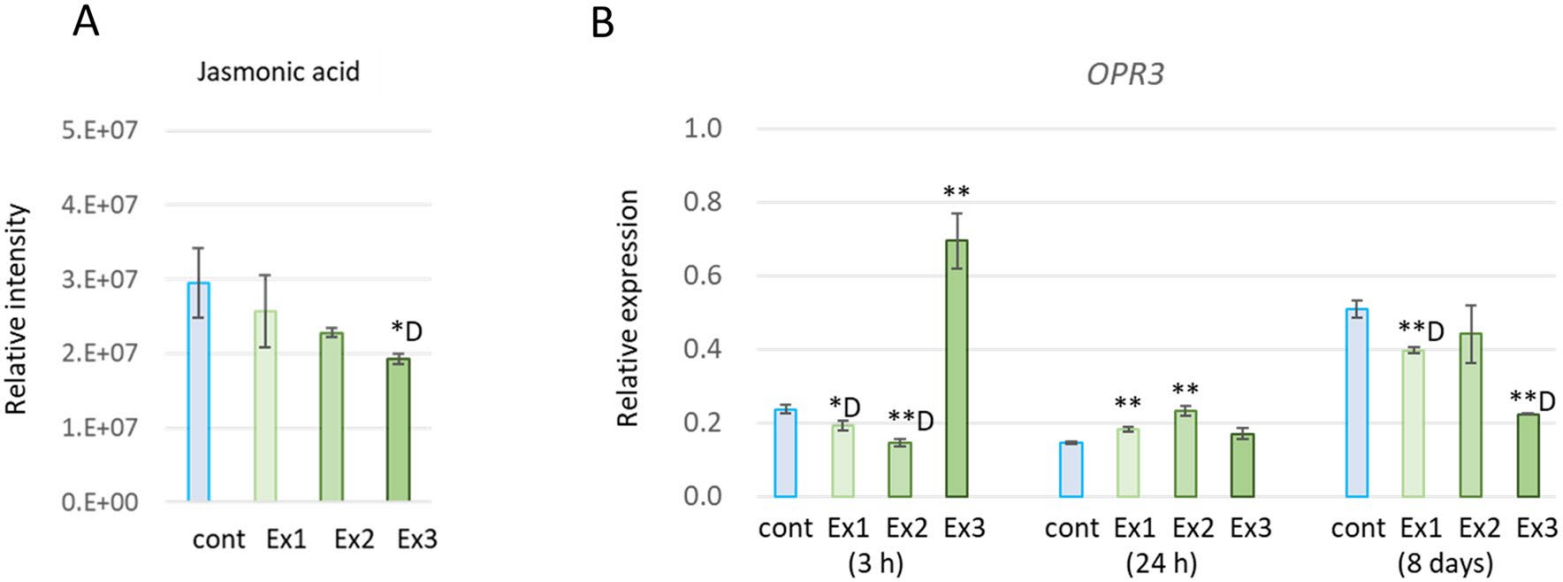
JA content and expression of JA biosynthesis gene *OPR3*. (**A**) JA was detected by non-targeted LC–MS. (**B**) Expression level of *OPR3* gene was analyzed by real-time RT-PCR. Data represent at least three biological samples in each metabolite and are mean ± S.E. Student’s *t-*test compared with each control level (*p < 0.05, **p < 0.01, D: downregulated).

Non-targeted LC–MS analysis also showed that endogenous Jasmonic acid (JA) content tended to decrease in a dose dependent manner with N_2_O_5_ exposure (Supplementary Table 1, Fig. 5A). Consistent with this, the expression of *OPR3* gene involved in JA biosynthesis was also significantly reduced in 32-day-old plant leaves under the same conditions, 8 days after the third exposure (Fig. 5B). On the other hand, in an experiment using 14-day-old plants to confirm the early responsiveness of gene expression, a significant increase in *OPR3* gene expression was observed 3 h after strong exposure to Ex3, while a slight but significant decrease was observed for Ex1 and Ex2. Conversely, a significant increasing trend was observed in Ex1 and Ex2 after 24 h, suggesting that the JA biosynthesis and response also respond transiently early after exposure.

## Discussion

APP technology enables powerful non-equilibrium chemical reactions initiated by energetic electrons, converting nitrogen (N_2_), oxygen (O_2_), and water (H_2_O) molecules into gaseous reactive species H_x_N_y_O_z_ [e.g., ozone (O_3_), nitric oxide (NO), nitrogen dioxide (NO_2_)]^19–21^. Its usefulness has been demonstrated in the fields of medicine^22–24^ and environmental science^25^, as well as in agricultural applications such as nitrogen fixation and plasma-treated water^26–28^. In particular, plant responses to gaseous O_3_, first reported by the APP technology in 1857^29^, have been intensively studied; O_3_ exposure induces systemic acquired resistance (SAR) via the salicylic acid system and promotes a hypersensitive response with program cell death^30,31^. On the other hand, N_2_O_5_, one of the reactive nitrogen species produced by our recent APP technology, has very unique properties in its effects on plants. It is highly water soluble, reacts with water and is efficiently converted to nitrate ions, which can be used as a nitrogen source for several plants^3^. Furthermore, exposure to N_2_O_5_ enhances plant immunity through increased secondary metabolites from tryptophan metabolism, but does not promote program cell death signaling in Arabidopsis^4^. We have also recently showed that in Arabidopsis, the systemic signaling response of JA, which is closely involved in the regulation of secondary metabolite biosynthesis, is immediately increased upon exposure to N_2_O_5_^5^. Taken together, exposure to N_2_O_5_ appears to induce systemic resistance (ISR) in plants that is different from O_3_-induced SAR.

Plant secondary metabolites have protective functions against biotic and abiotic stresses such as pathogen infections, wounds, UV radiation, oxidative damage, pollutants, and herbivores^32^. Of these, cineole and cinnamaldehyde have been reported to have strong repellent effects against aphids^33–35^. In this study, in sweet basil leaves exposed to N_2_O_5_ gas, terpenoids and phenylpropenes such as caryophyllene oxide, cinnamaldehyde, cineole, and methyl eugenol in the essential oil, coumarin and its derivatives such as esculetin and columbianetin, one of the phenylpropanoid chicoric acid, some flavonoids, and a lignan, were increased. Furthermore, the amount of increase in these secondary metabolites was found to depend on the amount of N_2_O_5_ exposure. At relatively low N_2_O_5_ exposure (Ex1), flavonoids such as kaempferol, quercetin, quercetin 3β-glucose and caryophyllene oxide were increased. At moderate exposure (Ex2), cineol and methyl eugenol were significantly increased. At the highest exposure (Ex3), plant fresh weight also decreased and cinnamaldehyde increased in a dose-dependent manner, while methyl cinnamate decreased inversely.

Interestingly, changes in the expression of enzyme genes involved in the biosynthesis of these secondary metabolites correlated well with the increase in metabolites due to N_2_O_5_ exposure. Among them, the cinnamoyl-CoA reductase *CCR* gene was suggested to be an important gene in the synthesis of secondary metabolites by N_2_O_5_ exposure, with its expression increasing even at low Ex1 exposure and reaching a plateau after 24 h and 8 days for Ex2 exposure (Fig. 4). CCR catalyzes the first reaction involved in the biosynthesis of plant lignin as well as essential oil monolignols and increases metabolic flux to well known to lead to flavonoids, stilbenes, hydroxycinnamic acids and their esters^36,37^. Under Ex1 conditions with low induction of *CCR* expression, flavonoid synthesis and a coumarin derivative columbianetic from coumaric acid may have been enhanced. The CCR accumulated by stronger N_2_O_5_ exposure (Ex3) may have been more reactive and converted cinnamic acid to cinnamaldehyde, resulting in a relative reduction in the synthesis of flavonoids and phenylpropanes (see Fig. 4). Many of the secondary metabolites found in this study have antioxidant, antibacterial, fungicidal, insecticidal and aphid repellent properties. These suggest that the level of plant defense response is progressively regulated in response to N_2_O_5_ exposure, resulting in changes in the production status of the respective secondary metabolites in sweet basil leaves.

In Arabidopsis, *AtCCR1* is ubiquitously expressed and *AtCCR2* expression is normally suppressed to low levels, and both are transiently induced by infection with the pathogen *Xanthomonas campestris* pv. *Campestris*^38^. In kenaf, *HcCCR2* expression is induced by several stress treatments such as wounding, salt, H_2_O_2_, and ABA, among which the highest expression induction is observed with methyl jasmonic acid (MeJA) treatment^36^. During in vitro ripening of *Fragaria chiloensis* fruit, MeJA treatment altered the expression levels of several phenylpropanoid pathway-related genes including *CCR*, *CAD* and chalcone isomerase^39^. JA widely promotes the synthesis of secondary metabolites such as lignin, terpenoids, phenylpropenes, or phenylpropanoids^7^. Indeed, plant essential oils obtained by foliar application of JA and MeJA have also been reported to increase methyl eugenol and other phenylpropanoids in basil leaves^40,41^. In this study, both the amount of endogenous JA and the expression level of the *OPR3* gene involved in JA biosynthesis decreased in a dose-dependent manner with N_2_O_5_ exposure in plant leaves 8 days after the third exposure (Fig. 5). On the other hand, the expression of the *OPR3* gene increased early after N_2_O_5_ exposure (3 and 24 h after the first exposure on 14-day-old plants), and similar early induction was observed for a group of other genes involved in the synthesis of secondary metabolites (Fig. 4). It has been reported that in Arabidopsis, that the amount of JA and the expression of its biosynthetic genes are significantly increased by insect feeding damage, but the peak is temporary and lasts from tens of minutes to several hours, after which it returns to the original level or to a lower level^42,43^. This means that the N_2_O_5_ exposure used in this study also induced a transient, non-sustained JA response, as seen in wounds, which in turn affected the synthesis of secondary metabolites. Indeed, l-glutathione GSH content, an antioxidant activated downstream of JA signaling^44^, tended to increase in a dose-dependent manner with N_2_O_5_ exposure.

**Figure 6:**
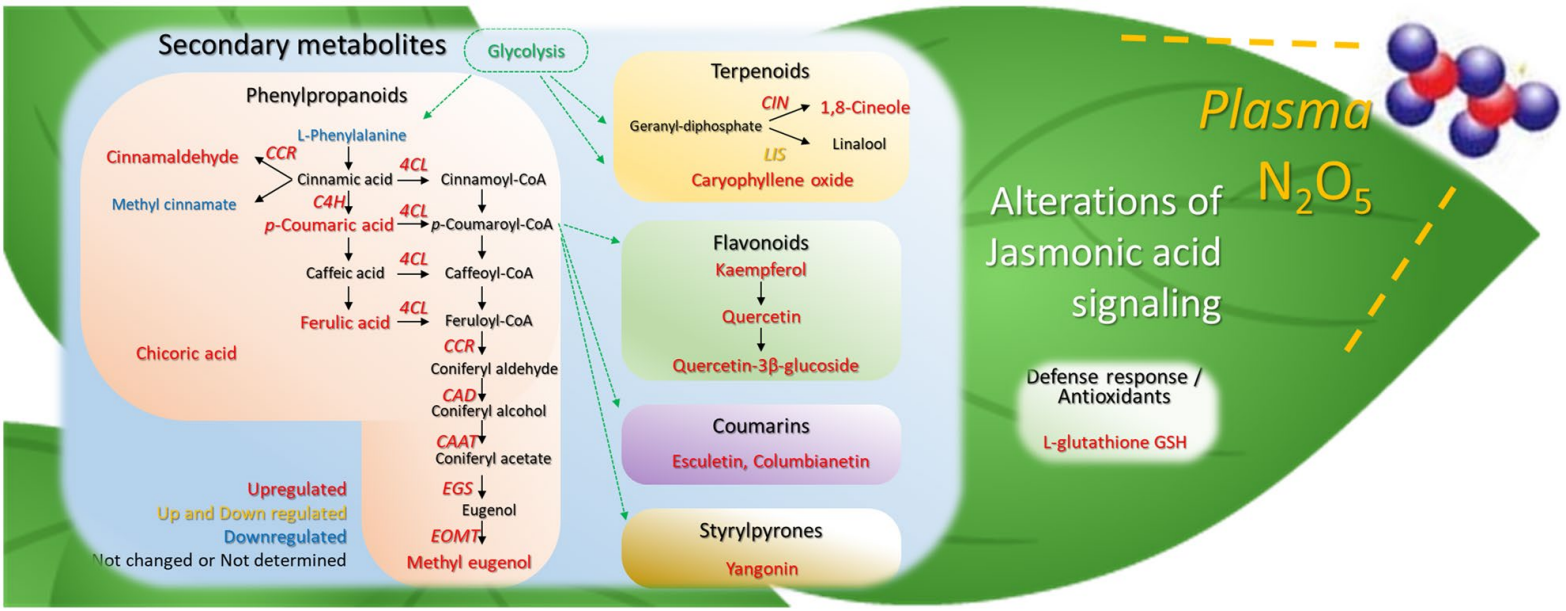
Schematic illustration of the increase of secondary metabolites in sweet basil leaves due to N_2_O_5_ exposure.

In conclusion, N_2_O_5_ gas application with APP-technology increased the production of highly functional plant secondary metabolites in sweet basil leaves via plant defense responses (Fig. 6). In previous studies, foliar application of MeJA and JA, as well as other abiotic stress treatments, have successfully increased or altered plant secondary metabolites^40,45–49^. On the other hand, exposure to N_2_O_5_ produced by APP is non-toxic and leaves no residues. Furthermore, the desired secondary metabolites can be increased by controlling the amount of N_2_O_5_ exposure. In sweet basil leaves, not only essential oil components but also 4-(3,4-dihydroxyphenyl)-6,7-dihydroxy-2-naphthoic acid and tanshinone IIA showed different increasing changes upon exposure to N_2_O_5_. These compounds are attracting attention as new therapeutic agents with antioxidant activity against reactive oxygen species in Alzheimer's disease^50–52^. Many of the secondary metabolites found in this study are also known to have antioxidant, anti-inflammatory and antiviral effects in humans. Furthermore, as N_2_O_5_ can be produced using only air and electricity, this new technology is expected to contribute to global environmental protection and bring innovation to agriculture and biotechnology.

## Methods

### N_2_O_5_ gas generation using APP technology

N_2_O_5_ gas, which cannot be stored in the atmosphere, was synthesized using a newly constructed portable device consisting of a plasma module, two mass flow controllers, electrical control components, and a mixing reactor, as described^1^. By mixing 1 L/min of NO_x_-rich plasma effluent gas and 1 L/min of O_3_-rich plasma effluent gas in a mixing reactor, 2 L/min of N_2_O_5_-rich plasma outflow gas can be generated. N_2_O_5_-rich gas used in the present study contained approximately 230 ppm of N_2_O_5_, corresponding to 0.32 μmol/s, with some impurities of around 30 ppm of O_3_ and NO_2_.

## Plant materials and growth conditions

Sweet basil (*O. basilicum* L.) cultivated seeds were purchased from Fujita Seed Co. (Osaka, Japan; Fujita’s original sweet basil). This basil produces methyl eugenol but not methyl chavicol. Three seeds of basil were sown in a seedling case (5 × 10 × 15 cm, Fujimoto Kagaku Co., Tokyo Japan) containing universal soil (KUMIAI NIPPI #1, Nippon Hiryo Co., Tokyo Japan). A total of six samples were used in two seedling cases per experimental condition. Plants were grown under natural light conditions in a net house at the Graduate School of Life Sciences, Tohoku University (Sendai, Japan) from May to June and watered every other day. Relative humidity during this period was approximately 70%. N_2_O_5_ gas exposures to sweet basil was carried out three times at three different doses at the following growth stages. The first N_2_O_5_ gas exposure was carried out on seedlings 14 days after germination (when secondary set of leaves had started to emerge). Each plant case was placed in a 60 L plastic bag and the bag was filled with N_2_O_5_ gas for 30 s (Ex1: 9.7 μmol), 60 s (Ex2: 19.4 μmol), and 90 s (Ex3: 29.1 μmol). Carefully closed and left for 3 min, plants were removed from the bag. A second similar N_2_O_5_ gas exposure was made 4 days later (plants were approximately 18 days old); a third exposure was performed 6 days later (plants were approximately 24 days old), and sampled 8 days after the third exposure (plants were approximately 32 days old plants and fifth set of leaves had started to emerge). Complete sets of expanded leaves from second to fifth order were collected, ground in liquid nitrogen using a mortar and pestle and used for gene expression, non-targeted LC–MS and targeted GC–MS analyses.

## RNA expression analysis

Total RNA was extracted from each 100 mg of ground frozen leaf tissue (14 and 32 days old plants) using TRI Reagent (Sigma-Aldrich). cDNA synthesis was performed using a Prime Script RT reagent Kit with gDNA Eraser (Takara Bio). The expression analysis of each target gene was performed quantitatively by real-time RT-PCR using TB Green Premix Ex TaqII (Tli RNasH Plus; Takara Bio) with Thermal cycler (CFX96, BioRad). The expression levels of the following genes were monitored with specific primer set of cinnamate 4-hydroxylase gene *C4H* (Accession No. HM990150)*,* 4-coumarate CoA ligase *4CL* (KC576841)*,* cinnamoyl-CoA reductase *CCR* (in this study)*,* cinnamyl alcohol dehydrogenase *CAD* (AY879285)*,* coniferyl alcohol acetyl transferase *CAAT1* (MN031888)*,* eugenol (and chavicol) synthase *EGS1* (DQ372812)*,* eugenol *O*-methyltransferase *EOMT* (AF435008, XLOC_068107 and XLOC_068808)*,* 1,8-cineole synthase *CIN* (in this study)*,* *S*-linalool synthase *LIS* (in this study)*, OPR3* (*O. basilicum* Chr: scaffold10, 7,268,155–7,270,534)*,* and *β-tubulin* (MH620961.1) genes in *O. basilicum* (Supplementary Table 2)^15–18^. Relative expression ratios for each target gene were calculated using *β-tubulin* gene expression in the same experimental samples. Basil *CCR, CIN* and *LIS* cDNA sequences were determined by RNA sequencing analysis in this study (Supplementary Table 2).

## Non-targeted metabolomic analysis of hydrophilic metabolites in basil leaves

Non-targeted metabolomic analysis of hydrophilic metabolites in basil leaves was carried out by Kazusa DNA Research Institute under contract (https://www.kazusa.or.jp/e/laboratories/ms_team.html)^53–55^. Untargeted metabolomic analysis of basil leaf sample was performed using an LC–MS system, UltiMate 3000 Rapid Separation LC (Thermo Fisher Scientific) and Q Exactive mass spectrometer (Thermo Fisher Scientific). Approximately 5 g of leaf sample in each condition (a set of expanded leaves from second to fifth order) was homogenized with 75% methanol and zirconia beads, and centrifuged at 20,000* g* for 10 min. The supernatant was filtered through a MonoSpin C18 column (GL Sciences, Tokyo, Japan), washed with 100% methanol, eluted with 75% methanol, and filtered through a 0.2 μm filter. LC separation was performed on an InterSustain AQ-C18 column (2.1 mm × 150 mm, particle size 3 μm, GL Sciences) with linear gradient of 0.1% (v/v) formic acid in water (A) and 0.1% (v/v) formic acid in acetonitrile (B). The flow rate was kept constant at 0.2 ml/min at 40 °C. The Q Exactive mass spectrometer was set to operate in the Electro Spray Ionization (ESI) mode. All spectra were acquired in the range of m/z 100–1,500. Full scan data were obtained at a resolution of 70,0000 and MS/MS data were obtained at a resolution of 17,5000 with data dependent scan (Top 10). The peaks were annotated using the Unique Connectivity of UnCharged compound (UC2) database25) and the ExactMassDB-HR2 (EX-HR2) database at the MFSearcher program26) with the predicted mass values of the original molecules at 5 ppm mass tolerance. The compound records of the UC2 database include the database records of the KNApSAcK27).

## Targeted-GC–MS analysis

The basis for the GC analysis of extracts from basil leaves was based on the following paper.^56^ To obtain a fraction containing the essential oil constituents of basil leaves, each 100 mg of ground frozen leaf tissue was mixed with 100 μl of ethyl acetate containing 180 ppm of toluene as an internal standard, vortexed for 1 min and centrifuged at 4℃, 15,000 rpm, for 5 min. The ethyl acetate layer was recovered and analyzed by GC–MS (Agilent 7890A GC and 5975C GC/MSD) with a DB-1 capillary column (30 m × 0.25 mm × 0.25 μm; J and W Scientific). The column temperature was increased from 55 to 210℃ at a rate of 20℃/min, and held for 4 min. In the analysis, nitrogen gas was used as a carrier gas in splitless mode, with a flow rate of 1 mL/min. The SIM mode was used to detect each compound, and the acquisition time windows and monitored ions are shown in Table 1. To increase the sensitivity, the Gain Factor was set to its maximum value of 25. For quantification of each compound, cineole, linalool, methyl chavicol, eugenol and methyl eugenol (Sigma-Aldrich), standard solutions containing 25, 50, 100, 250, 500, and 1000 ppm were injected.

**Table 1:** Detection conditions for targeted GC–MS analysis.

Compounds	Time windows (min)	Monitored ions (m/z)
Toluene	3.25–4.69	65, 91, 92
Cineole	10.90–11.79	43, 81, 108
Linalool	11.80–12.69	55, 71, 93
Methyl chavicol	12.70–14.09	117, 121, 148
Eugenol	14.10–15.99	91, 103, 149, 164, 178
Methyl eugenol		

## Quantification and statistical analysis

All analyses were performed in at least biological triplicate for each sample. Statistical analyses were performed using unequal variance *t*-test. *p* values less than 0.05 were classed as statistically significant.

### Supplementary Information

Supplementary Table 1.

Supplementary Table 2.

## Data Availability

All data is provided within the main body and the Supplementary information (SI). Other data that support the findings of this study are available from the corresponding author upon reasonable request.
