# Supplementary material for: Increase of secondary metabolites in sweet basil (Ocimum basilicum L.) leaves by exposure to N2O5 with plasma technology
Source: Sci Rep. 2024 Jun 4;14:12759. doi: 10.1038/s41598-024-63508-8 (PMC11150270; doi:10.1038/s41598-024-63508-8)
Supplement: Supplementary file 1 — Supplementary Table 1. [file 41598_2024_63508_MOESM1_ESM.docx]

Supplementary Table 1. Exposure to N_2_O_5_ altered 38 of 151 compounds identified by non-targeted LC-MS in sweet basil leaves.

| **R.T. (min)** | **Formula** | **Metabolite Name (Click to View Detail)** | **cont (Ave.** | **± SE)** | **Ex1 (Ave.** | **± SE)** | ***p* value** | **Ex2 (Ave.** | **± SE)** | ***p* value** | **Ex3 (Ave.** | **± SE)** | ***p* value** |
| --- | --- | --- | --- | --- | --- | --- | --- | --- | --- | --- | --- | --- | --- |
| 3.10 | C7H14N2O3 | [N-Acetylornithine](https://www.mzcloud.org/compound/reference/513) | **8.4E+06** | 5.1E+06 | **1.3E+06** | 1.5E+05 | *0.16* | **7.1E+05** | 2.5E+04 | *0.14* | **6.5E+05** | 2.0E+04 | *0.14* |
| 3.17 | C6H11NO2 | [Pipecolic acid](https://www.mzcloud.org/compound/reference/548) | **3.1E+07** | 4.6E+06 | **4.7E+07** | 9.0E+06 | *0.13* | **4.5E+07** | 1.2E+07 | *0.21* | **4.2E+07** | 6.3E+06 | *0.15* |
| 3.32 | C8H16N2O3 | [N6-Acetyl-L-lysine](https://www.mzcloud.org/compound/reference/501) | **1.3E+07** | 3.8E+06 | **1.3E+07** | 2.3E+06 | *0.48* | **8.1E+06** | 7.8E+05 | *0.19* | **4.9E+06** | 3.8E+05 | *0.08* |
| 3.33 | C10H14N5O7P | [Adenosine 5'-monophosphate](https://www.mzcloud.org/compound/reference/252) | **1.1E+08** | 8.4E+06 | **1.1E+08** | 7.3E+06 | *0.26* | **1.2E+08** | 1.1E+07 | *0.22* | **1.0E+08** | 5.1E+06 | *0.39* |
| 3.33 | C8H16N4O3 | [Acetylarginine](https://www.mzcloud.org/compound/reference/4035) | **2.5E+07** | 2.6E+06 | **2.5E+07** | 1.4E+06 | *0.45* | **2.5E+07** | 6.6E+05 | *0.45* | **2.4E+07** | 9.9E+05 | *0.38* |
| 3.33 | C10H18N4O6 | [Argininosuccinic acid](https://www.mzcloud.org/compound/reference/324) | **1.5E+07** | 7.5E+06 | **1.5E+07** | 3.4E+06 | *0.50* | **3.4E+07** | 5.1E+06 | *0.08* | **1.2E+07** | 1.6E+06 | *0.36* |
| 3.34 | C21H27N7O14P2 | * [Nicotinamide adenine dinucleotide (NAD+)](https://www.mzcloud.org/compound/reference/873) | **5.2E+07** | 2.6E+06 | **5.2E+07** | 2.3E+06 | *0.49* | **5.6E+07** | 1.9E+06 | *0.19* | **6.6E+07** | 2.5E+06 | *0.02* |
| 3.35 | C8H9NO3 | [2-Amino-3-methoxybenzoic acid](https://www.mzcloud.org/compound/reference/1726) | **5.4E+06** | 1.0E+06 | **9.2E+06** | 3.6E+06 | *0.23* | **4.0E+06** | 4.3E+05 | *0.17* | **5.6E+06** | 5.6E+05 | *0.44* |
| 3.46 | C5H9NO4 | [L-Glutamic acid](https://www.mzcloud.org/compound/reference/470) | **4.8E+06** | 9.9E+05 | **1.1E+07** | 5.3E+06 | *0.19* | **9.9E+06** | 2.5E+06 | *0.10* | **4.2E+06** | 3.9E+05 | *0.33* |
| 3.49 | C8H10NO6P | [Pyridoxal 5'-phosphate](https://www.mzcloud.org/compound/reference/1266) | **1.3E+07** | 2.3E+06 | **1.7E+07** | 2.4E+06 | *0.21* | **1.6E+07** | 2.0E+06 | *0.22* | **1.3E+07** | 1.6E+06 | *0.49* |
| 3.59 | C10H18N2O3 | [Valylproline](https://www.mzcloud.org/compound/reference/773) | **2.4E+07** | 1.4E+06 | **2.2E+07** | 9.9E+05 | *0.21* | **2.1E+07** | 9.1E+05 | *0.13* | **2.1E+07** | 1.7E+06 | *0.15* |
| 3.81 | C5H7NO3 | [D-(+)-Pyroglutamic Acid](https://www.mzcloud.org/compound/reference/417) | **1.7E+08** | 4.3E+07 | **1.9E+08** | 1.4E+07 | *0.36* | **2.9E+08** | 4.0E+07 | *0.08* | **1.9E+08** | 4.8E+07 | *0.40* |
| 3.88 | C5H4N4O | [Hypoxanthine](https://www.mzcloud.org/compound/reference/441) | **3.1E+07** | 1.2E+07 | **2.2E+08** | 9.4E+07 | *0.09* | **2.8E+07** | 6.6E+06 | *0.44* | **1.6E+07** | 1.2E+06 | *0.20* |
| 3.99 | C6H11NO4 | [Methyl 2-[(2-methoxy-2-oxoethyl)amino]acetate](https://www.mzcloud.org/compound/reference/4223) | **1.7E+07** | 2.7E+06 | **1.7E+07** | 2.5E+06 | *0.49* | **1.4E+07** | 1.6E+06 | *0.29* | **1.3E+07** | 1.1E+06 | *0.18* |
| 4.32 | C6H7N5O | [1-Methylguanine](https://www.mzcloud.org/compound/reference/104) | **5.1E+07** | 4.8E+06 | **5.0E+07** | 2.7E+06 | *0.43* | **4.6E+07** | 1.5E+06 | *0.22* | **4.8E+07** | 1.4E+06 | *0.35* |
| 4.52 | C9H8O3 | [2-Hydroxycinnamic acid](https://www.mzcloud.org/compound/reference/168) | **6.7E+07** | 1.1E+07 | **6.0E+07** | 1.7E+06 | *0.31* | **7.2E+07** | 6.1E+06 | *0.37* | **4.7E+07** | 1.7E+06 | *0.10* |
| 4.54 | C5H10N2O3 | [DL-Glutamine](https://www.mzcloud.org/compound/reference/2967) | **1.2E+07** | 2.7E+06 | **9.7E+06** | 6.5E+05 | *0.30* | **1.2E+07** | 1.6E+06 | *0.50* | **8.7E+06** | 1.1E+06 | *0.23* |
| 4.54 | C4H4N2O2 | [Uracil](https://www.mzcloud.org/compound/reference/2531) | **2.0E+07** | 2.9E+06 | **2.6E+07** | 5.6E+06 | *0.23* | **2.9E+07** | 3.5E+06 | *0.09* | **2.9E+07** | 2.4E+06 | *0.06* |
| 4.75 | C6H13NO2 | [L-Isoleucine](https://www.mzcloud.org/compound/reference/2244) | **1.5E+09** | 3.1E+08 | **1.5E+09** | 1.5E+08 | *0.48* | **2.2E+09** | 3.3E+08 | *0.14* | **1.2E+09** | 5.6E+07 | *0.20* |
| 4.94 | C7H6O2 | [4-Hydroxybenzaldehyde](https://www.mzcloud.org/compound/reference/234) | **7.2E+07** | 1.0E+07 | **6.6E+07** | 2.3E+06 | *0.32* | **7.8E+07** | 7.2E+06 | *0.38* | **5.7E+07** | 1.2E+06 | *0.14* |
| 4.94 | C9H11NO3 | [L-Tyrosine](https://www.mzcloud.org/compound/reference/2255) | **7.0E+08** | 1.0E+08 | **6.3E+08** | 2.3E+07 | *0.30* | **7.4E+08** | 6.8E+07 | *0.40* | **5.5E+08** | 1.2E+07 | *0.14* |
| 4.94 | C8H9NO | [N-Benzylformamide](https://www.mzcloud.org/compound/reference/817) | **2.6E+08** | 3.6E+07 | **2.3E+08** | 7.7E+06 | *0.30* | **2.7E+08** | 2.3E+07 | *0.41* | **2.0E+08** | 4.5E+06 | *0.14* |
| 4.96 | C12H20N2O3 | [Pirbuterol](https://www.mzcloud.org/compound/reference/2368) | **3.3E+07** | 2.6E+06 | **3.1E+07** | 4.0E+06 | *0.37* | **3.3E+07** | 3.6E+06 | *0.50* | **3.2E+07** | 6.0E+06 | *0.48* |
| 5.15 | C14H20N6O5S | [S-Adenosylhomocysteine](https://www.mzcloud.org/compound/reference/592) | **4.5E+06** | 3.3E+05 | **4.6E+06** | 1.2E+05 | *0.43* | **5.6E+06** | 3.7E+05 | *0.07* | **5.2E+06** | 5.8E+05 | *0.21* |
| 5.21 | C6H13NO2 | [Leucine](https://www.mzcloud.org/compound/reference/6) | **1.9E+09** | 4.2E+08 | **2.0E+09** | 3.1E+08 | *0.44* | **3.1E+09** | 4.8E+08 | *0.11* | **1.5E+09** | 1.5E+08 | *0.21* |
| 5.48 | C6H7N5O | [7-Methylguanine](https://www.mzcloud.org/compound/reference/276) | **9.0E+07** | 5.5E+06 | **8.7E+07** | 8.6E+05 | *0.33* | **8.9E+07** | 3.0E+06 | *0.46* | **8.4E+07** | 4.8E+06 | *0.28* |
| 5.50 | C8H11NO | [Tyramine](https://www.mzcloud.org/compound/reference/1758) | **9.5E+07** | 9.0E+06 | **1.3E+08** | 1.3E+07 | *0.06* | **9.0E+07** | 8.8E+06 | *0.39* | **9.0E+07** | 5.9E+06 | *0.39* |
| 6.48 | C10H13N5O4 | [Adenosine](https://www.mzcloud.org/compound/reference/297) | **4.6E+08** | 9.5E+07 | **6.2E+08** | 1.1E+08 | *0.22* | **6.1E+08** | 4.9E+07 | *0.16* | **6.0E+08** | 3.4E+07 | *0.16* |
| 6.65 | C6H11NO3 | [4-Acetamidobutanoic acid](https://www.mzcloud.org/compound/reference/7610) | **8.5E+06** | 3.1E+06 | **2.0E+07** | 5.3E+06 | *0.10* | **4.7E+06** | 1.3E+06 | *0.20* | **1.9E+06** | 4.1E+05 | *0.08* |
| 6.75 | C6H6N4O | [6-Methyl[1,2,4]triazolo[4,3-b]pyridazin-8-ol](https://www.mzcloud.org/compound/reference/4332) | **7.3E+06** | 4.4E+05 | **7.3E+06** | 7.1E+05 | *0.48* | **8.1E+06** | 4.3E+05 | *0.19* | **7.7E+06** | 3.6E+05 | *0.30* |
| 7.17 | C10H12N4O5 | [Inosine](https://www.mzcloud.org/compound/reference/1234) | **5.6E+06** | 1.2E+06 | **9.1E+06** | 3.3E+06 | *0.23* | **1.1E+07** | 2.6E+06 | *0.10* | **7.3E+06** | 1.0E+06 | *0.20* |
| 7.35 | C5H5N5O | * [Guanine](https://www.mzcloud.org/compound/reference/436) | **5.6E+07** | 4.4E+06 | **5.3E+07** | 4.7E+06 | *0.34* | **7.7E+07** | 1.1E+06 | *0.01* | **6.6E+07** | 3.7E+06 | *0.11* |
| 8.60 | C6H11NO2 | [Vigabatrin](https://www.mzcloud.org/compound/reference/3554) | **3.3E+08** | 1.3E+08 | **6.8E+08** | 1.1E+08 | *0.08* | **1.4E+08** | 1.2E+07 | *0.14* | **3.9E+07** | 1.3E+07 | *0.06* |
| 8.88 | C14H22N2O4 | [2-(3-{[3-(Tetrahydro-2H-pyran-4-ylamino)-3-oxetanyl]methyl}-1,2-oxazol-5-yl)ethanol](https://www.mzcloud.org/compound/reference/10173) | **2.3E+06** | 1.5E+06 | **2.1E+06** | 8.8E+05 | *0.47* | **2.9E+06** | 1.1E+06 | *0.40* | **7.2E+05** | 3.0E+05 | *0.23* |
| 8.90 | C7H6O3 | [4-Hydroxybenzoic acid](https://www.mzcloud.org/compound/reference/148) | **2.1E+07** | 2.7E+06 | **2.2E+07** | 2.9E+06 | *0.38* | **1.7E+07** | 1.1E+06 | *0.20* | **1.6E+07** | 1.4E+06 | *0.15* |
| 9.05 | C9H10N2O | ** [1-(1H-Benzo[d]imidazol-2-yl)ethan-1-ol](https://www.mzcloud.org/compound/reference/6534) | **9.1E+06** | 9.6E+05 | **9.0E+06** | 1.4E+06 | *0.47* | **5.7E+06** | 6.0E+05 | *0.03* | **5.1E+06** | 4.0E+05 | *0.02* |
| 9.21 | C9H11NO2 | **[L-Phenylalanine](https://www.mzcloud.org/compound/reference/8) | **2.0E+09** | 5.7E+08 | **1.8E+09** | 1.3E+08 | *0.40* | **1.9E+09** | 3.5E+08 | *0.44* | **8.7E+08** | 4.1E+07 | *0.00* |
| 9.39 | C5H10N2O | [1-Nitrosopiperidine (NPIP)](https://www.mzcloud.org/compound/reference/3370) | **4.5E+08** | 3.7E+07 | **4.0E+08** | 5.0E+07 | *0.31* | **3.8E+08** | 1.9E+07 | *0.12* | **3.7E+08** | 2.4E+07 | *0.12* |
| 9.90 | C9H6O3 | [7-Hydroxycoumarine](https://www.mzcloud.org/compound/reference/1341) | **4.0E+06** | 4.4E+05 | **3.4E+06** | 2.3E+05 | *0.16* | **4.0E+06** | 4.5E+05 | *0.47* | **6.5E+06** | 1.2E+06 | *0.10* |
| 10.17 | C10H9NO3 | ** [5-Hydroxyindole-3-acetic acid](https://www.mzcloud.org/compound/reference/256) | **8.4E+06** | 1.7E+05 | **8.2E+06** | 2.0E+05 | *0.32* | **8.7E+06** | 2.7E+05 | *0.28* | **7.2E+06** | 4.1E+05 | *0.05* |
| 10.40 | C6H14O3 | ** [Trimethylolpropane](https://www.mzcloud.org/compound/reference/2684) | **2.4E+07** | 6.0E+05 | **2.4E+07** | 7.5E+05 | *0.31* | **2.3E+07** | 1.4E+06 | *0.22* | **2.0E+07** | 1.5E+06 | *0.05* |
| 10.44 | C10H7NO4 | [Xanthurenic acid](https://www.mzcloud.org/compound/reference/789) | **6.4E+06** | 1.5E+06 | **9.1E+06** | 1.2E+06 | *0.16* | **7.1E+06** | 4.4E+05 | *0.37* | **1.0E+07** | 1.2E+06 | *0.08* |
| 10.56 | C11H20N2O3 | [Leucylproline](https://www.mzcloud.org/compound/reference/468) | **8.2E+06** | 1.3E+06 | **8.8E+06** | 8.2E+05 | *0.38* | **7.3E+06** | 3.3E+05 | *0.29* | **6.4E+06** | 4.5E+05 | *0.16* |
| 10.68 | C14H17N5O3 | [Pipemidic acid](https://www.mzcloud.org/compound/reference/1672) | **3.1E+06** | 1.1E+06 | **8.6E+06** | 2.6E+06 | *0.09* | **2.8E+06** | 1.8E+05 | *0.43* | **8.2E+05** | 6.9E+04 | *0.09* |
| 10.87 | C9H9NO3 | ** [3-Succinoylpyridine](https://www.mzcloud.org/compound/reference/220) | **8.5E+06** | 2.2E+06 | **4.3E+06** | 4.4E+05 | *0.10* | **2.2E+06** | 4.7E+05 | *0.04* | **1.3E+06** | 1.7E+05 | *0.03* |
| 10.90 | C11H15N5O3S | [5'-S-Methyl-5'-thioadenosine](https://www.mzcloud.org/compound/reference/253) | **2.1E+08** | 4.7E+07 | **2.6E+08** | 1.6E+07 | *0.24* | **2.2E+08** | 7.6E+06 | *0.47* | **1.7E+08** | 8.3E+06 | *0.24* |
| 10.97 | C9H10O3 | [Apocynin](https://www.mzcloud.org/compound/reference/5847) | **4.1E+06** | 7.7E+05 | **5.0E+06** | 2.7E+05 | *0.21* | **3.8E+06** | 4.7E+05 | *0.41* | **5.1E+06** | 5.3E+05 | *0.21* |
| 11.08 | C11H12N2O2 | [DL-Tryptophan](https://www.mzcloud.org/compound/reference/415) | **6.7E+08** | 1.6E+08 | **5.9E+08** | 1.8E+07 | *0.35* | **5.1E+08** | 9.8E+07 | *0.28* | **3.0E+08** | 3.7E+07 | *0.07* |
| 11.23 | C9H10O5 | [Syringic acid](https://www.mzcloud.org/compound/reference/1269) | **9.1E+07** | 2.7E+07 | **1.6E+08** | 3.7E+07 | *0.14* | **1.5E+08** | 3.3E+07 | *0.16* | **1.1E+08** | 2.6E+07 | *0.39* |
| 11.32 | C9H6O4 | * [Esculetin](https://www.mzcloud.org/compound/reference/3417) | **4.8E+06** | 4.7E+05 | **7.8E+06** | 1.1E+06 | *0.06* | **9.4E+06** | 1.1E+06 | *0.02* | **8.6E+06** | 5.1E+05 | *0.01* |
| 11.35 | C9H10O | [2,4-Dimethylbenzaldehyde](https://www.mzcloud.org/compound/reference/6558) | **1.3E+07** | 1.6E+06 | **1.4E+07** | 1.3E+06 | *0.37* | **1.4E+07** | 1.2E+06 | *0.47* | **9.2E+06** | 5.0E+05 | *0.06* |
| 11.36 | C9H10O2 | ** [4'-Methoxyacetophenone](https://www.mzcloud.org/compound/reference/6574) | **7.6E+06** | 7.0E+05 | **7.2E+06** | 5.0E+05 | *0.38* | **7.3E+06** | 5.1E+05 | *0.39* | **5.4E+06** | 3.3E+05 | *0.04* |
| 11.52 | C10H7NO3 | [Kynurenic acid](https://www.mzcloud.org/compound/reference/458) | **2.0E+08** | 7.1E+06 | **2.0E+08** | 8.7E+06 | *0.33* | **2.0E+08** | 8.5E+06 | *0.34* | **1.9E+08** | 1.1E+07 | *0.40* |
| 11.54 | C17H21N4O9P | [Flavin mononucleotide (FMN)](https://www.mzcloud.org/compound/reference/589) | **1.4E+07** | 9.0E+05 | **1.3E+07** | 6.0E+05 | *0.36* | **1.9E+07** | 2.4E+06 | *0.07* | **1.6E+07** | 8.0E+05 | *0.12* |
| 11.58 | C9H7NO | [8-Hydroxyquinoline](https://www.mzcloud.org/compound/reference/2633) | **4.2E+06** | 1.2E+06 | **1.1E+07** | 3.3E+06 | *0.10* | **3.7E+06** | 4.6E+05 | *0.37* | **2.8E+06** | 1.2E+05 | *0.18* |
| 11.75 | C14H18N2O5 | [Aspartame](https://www.mzcloud.org/compound/reference/990) | **9.2E+06** | 2.5E+06 | **1.0E+07** | 3.2E+05 | *0.40* | **7.8E+06** | 3.3E+05 | *0.34* | **4.5E+06** | 3.6E+05 | *0.10* |
| 11.77 | C11H12O3 | [BMK methyl glycidate](https://www.mzcloud.org/compound/reference/6496) | **1.2E+07** | 8.2E+05 | **1.2E+07** | 6.5E+05 | *0.47* | **1.1E+07** | 6.6E+05 | *0.22* | **1.0E+07** | 2.6E+05 | *0.07* |
| 11.87 | C15H22N4O3 | [(S)-N-α-benzoylarginine ethyl ester](https://www.mzcloud.org/compound/reference/4188) | **6.2E+06** | 1.6E+06 | **5.4E+06** | 5.6E+05 | *0.37* | **4.7E+06** | 1.3E+06 | *0.30* | **7.6E+06** | 1.4E+06 | *0.31* |
| 11.88 | C10H12O2 | [4-Phenylbutyric acid](https://www.mzcloud.org/compound/reference/3074) | **2.4E+07** | 1.6E+06 | **2.6E+07** | 1.5E+06 | *0.30* | **2.2E+07** | 7.8E+05 | *0.16* | **2.1E+07** | 1.4E+06 | *0.11* |
| 11.96 | C11H12O5 | [Sinapinic acid](https://www.mzcloud.org/compound/reference/1192) | **2.7E+07** | 3.3E+06 | **2.9E+07** | 6.8E+06 | *0.41* | **2.3E+07** | 3.9E+06 | *0.27* | **3.2E+07** | 7.4E+06 | *0.32* |
| 12.05 | C7H6O3 | ** [3,4-Dihydroxybenzaldehyde](https://www.mzcloud.org/compound/reference/3393) | **2.4E+07** | 2.3E+06 | **2.4E+07** | 2.6E+06 | *0.45* | **1.7E+07** | 1.1E+06 | *0.06* | **1.3E+07** | 1.1E+06 | *0.01* |
| 12.16 | C22H18O12 | * [Chicoric acid](https://www.mzcloud.org/compound/reference/7791) | **3.0E+07** | 5.8E+06 | **5.4E+07** | 6.6E+06 | *0.05* | **5.0E+07** | 2.5E+06 | *0.03* | **4.9E+07** | 2.3E+06 | *0.03* |
| 12.30 | C27H30O15 | [5,7-Dihydroxy-2-(4-hydroxyphenyl)-6,8-bis[3,4,5-trihydroxy-6-(hydroxymethyl)tetrahydro-2H-pyran-2-yl]-4H-chromen-4-one](https://www.mzcloud.org/compound/reference/8227) | **1.4E+07** | 1.6E+06 | **3.6E+07** | 1.1E+07 | *0.10* | **2.0E+07** | 5.7E+06 | *0.20* | **1.3E+07** | 3.5E+06 | *0.43* |
| 12.38 | C19H30O8 | [3-Hydroxy-3,5,5-trimethyl-4-(3-oxo-1-buten-1-ylidene)cyclohexyl β-D-glucopyranoside](https://www.mzcloud.org/compound/reference/8213) | **8.2E+06** | 1.3E+06 | **8.1E+06** | 8.9E+05 | *0.48* | **8.2E+06** | 9.1E+05 | *0.49* | **6.5E+06** | 1.0E+06 | *0.21* |
| 12.55 | C14H14O4 | [(5S,6S)-5-Hydroxy-4-methoxy-6-[(E)-2-phenylvinyl]-5,6-dihydro-2H-pyran-2-one](https://www.mzcloud.org/compound/reference/7839) | **9.9E+06** | 2.3E+06 | **7.9E+06** | 1.3E+05 | *0.25* | **7.3E+06** | 8.2E+05 | *0.21* | **5.7E+06** | 1.3E+05 | *0.10* |
| 12.61 | C9H11NO4 | [L-Dopa](https://www.mzcloud.org/compound/reference/14) | **3.7E+07** | 1.7E+07 | **5.6E+07** | 4.3E+06 | *0.21* | **4.0E+07** | 3.9E+06 | *0.45* | **3.2E+07** | 1.3E+06 | *0.41* |
| 12.61 | C18H17NO7 | [trans-Clovamide](https://www.mzcloud.org/compound/reference/6156) | **9.3E+07** | 4.2E+07 | **1.4E+08** | 9.9E+06 | *0.20* | **1.0E+08** | 1.3E+07 | *0.42* | **8.0E+07** | 3.3E+06 | *0.41* |
| 12.64 | C10H18O4 | [Sebacic acid](https://www.mzcloud.org/compound/reference/916) | **1.1E+07** | 5.1E+05 | **1.0E+07** | 4.0E+05 | *0.35* | **1.1E+07** | 4.8E+05 | *0.35* | **9.9E+06** | 3.0E+05 | *0.18* |
| 12.67 | C12H18O3 | Jasmonic acid | **3.0E+07** | 4.7E+06 | **2.6E+07** | 4.8E+06 | *0.33* | **2.3E+07** | 6.8E+05 | *0.16* | **1.9E+07** | 6.8E+05 | *0.08* |
| 12.75 | C10H12O | ** [Cuminaldehyde](https://www.mzcloud.org/compound/reference/359) | **5.1E+07** | 4.2E+06 | **5.3E+07** | 2.6E+06 | *0.36* | **4.7E+07** | 3.1E+06 | *0.28* | **3.6E+07** | 1.4E+06 | *0.02* |
| 12.78 | C10H10O2 | [Methyl cinnamate](https://www.mzcloud.org/compound/reference/6564) | **1.4E+07** | 2.6E+06 | **1.2E+07** | 9.1E+05 | *0.27* | **8.1E+06** | 1.1E+06 | *0.09* | **7.6E+06** | 5.8E+05 | *0.07* |
| 13.08 | C15H12O6 | [Eriodictyol](https://www.mzcloud.org/compound/reference/411) | **8.0E+06** | 2.2E+06 | **2.9E+07** | 8.8E+06 | *0.07* | **1.6E+07** | 5.4E+06 | *0.15* | **1.3E+07** | 5.2E+06 | *0.24* |
| 13.12 | C10H17N3O6S | * [L-Glutathione (reduced)](https://www.mzcloud.org/compound/reference/472) | **1.7E+07** | 1.3E+06 | **2.1E+07** | 2.2E+06 | *0.10* | **2.2E+07** | 3.0E+06 | *0.12* | **3.6E+07** | 1.2E+06 | *0.00* |
| 13.29 | C15H20O3 | [(3aR,8R,8aR,9aR)-8-Hydroxy-8a-methyl-3,5-bis(methylene)decahydronaphtho[2,3-b]furan-2(3H)-one](https://www.mzcloud.org/compound/reference/7684) | **1.9E+07** | 4.4E+06 | **1.6E+07** | 2.3E+06 | *0.33* | **1.1E+07** | 4.8E+05 | *0.12* | **9.0E+06** | 4.8E+05 | *0.07* |
| 13.52 | C15H10O6 | [Luteolin](https://www.mzcloud.org/compound/reference/1316) | **5.8E+07** | 1.2E+07 | **1.2E+08** | 3.5E+07 | *0.12* | **8.1E+07** | 1.4E+07 | *0.18* | **6.5E+07** | 1.7E+07 | *0.39* |
| 13.81 | C15H10O7 | * [Quercetin](https://www.mzcloud.org/compound/reference/27) | **3.5E+06** | 7.3E+05 | **6.6E+06** | 8.2E+05 | *0.04* | **6.4E+06** | 1.0E+06 | *0.07* | **4.2E+06** | 4.6E+05 | *0.29* |
| 13.81 | C11H8O4 | [3,5-Dihydroxy-2-naphthoic acid](https://www.mzcloud.org/compound/reference/619) | **1.5E+07** | 5.5E+06 | **7.9E+07** | 3.6E+07 | *0.12* | **3.0E+07** | 5.3E+06 | *0.10* | **1.3E+07** | 4.5E+06 | *0.40* |
| 13.84 | C7H10O2 | [β,β-Dimethyl-γ-methylene-γ-butyrolactone](https://www.mzcloud.org/compound/reference/2631) | **4.2E+06** | 1.6E+05 | **4.2E+06** | 1.7E+05 | *0.48* | **4.3E+06** | 2.2E+05 | *0.39* | **4.1E+06** | 3.7E+05 | *0.45* |
| 13.86 | C15H15NO4 | [6-(Allyloxy)-1-ethyl-4-oxo-1,4-dihydroquinoline-2-carboxylic acid](https://www.mzcloud.org/compound/reference/8720) | **1.1E+07** | 6.0E+06 | **5.4E+06** | 5.2E+05 | *0.25* | **2.3E+06** | 9.6E+05 | *0.16* | **6.2E+05** | 2.5E+05 | *0.12* |
| 14.12 | C22H33N5O4 | [3-{(4R,7S,8aS)-7-[(Isopropylcarbamoyl)amino]-1-oxooctahydropyrrolo[1,2-a]pyrazin-4-yl}-N-(4-methoxybenzyl)propanamide](https://www.mzcloud.org/compound/reference/9225) | **4.1E+06** | 1.0E+06 | **4.4E+06** | 8.8E+05 | *0.44* | **4.1E+06** | 7.3E+05 | *0.50* | **1.9E+06** | 3.1E+05 | *0.09* |
| 14.13 | C8H16O | [Sulcatol](https://www.mzcloud.org/compound/reference/2606) | **1.4E+07** | 4.5E+06 | **9.0E+06** | 3.4E+05 | *0.23* | **6.2E+06** | 1.0E+06 | *0.13* | **9.9E+06** | 2.2E+06 | *0.29* |
| 14.30 | C21H20O12 | * [Quercetin-3β-D-glucoside](https://www.mzcloud.org/compound/reference/1472) | **1.5E+07** | 3.0E+06 | **2.8E+07** | 3.3E+06 | *0.04* | **2.5E+07** | 1.8E+06 | *0.04* | **2.3E+07** | 2.9E+06 | *0.10* |
| 14.43 | C15H10O5 | [Apigenin](https://www.mzcloud.org/compound/reference/20) | **1.2E+07** | 2.4E+06 | **2.6E+07** | 8.2E+06 | *0.13* | **1.7E+07** | 3.6E+06 | *0.17* | **1.5E+07** | 4.3E+06 | *0.29* |
| 14.62 | C10H16O | [α-Pinene-2-oxide](https://www.mzcloud.org/compound/reference/323) | **7.6E+08** | 2.2E+07 | **7.0E+08** | 1.1E+08 | *0.34* | **7.1E+08** | 8.4E+07 | *0.36* | **6.7E+08** | 3.1E+07 | *0.07* |
| 14.64 | C9H9N | [3-Phenylpropionitrile](https://www.mzcloud.org/compound/reference/625) | **5.5E+06** | 4.7E+05 | **5.4E+06** | 1.2E+06 | *0.46* | **4.5E+06** | 4.0E+05 | *0.12* | **4.7E+06** | 2.5E+05 | *0.14* |
| 15.01 | C15H10O6 | * [Kaempferol](https://www.mzcloud.org/compound/reference/966) | **6.2E+06** | 1.0E+06 | **1.4E+07** | 1.2E+06 | *0.01* | **6.8E+06** | 7.5E+05 | *0.36* | **1.1E+07** | 2.5E+04 | *0.01* |
| 15.11 | C10H8O4 | [Scopoletin](https://www.mzcloud.org/compound/reference/970) | **3.8E+06** | 1.3E+06 | **4.4E+06** | 5.1E+05 | *0.37* | **2.5E+06** | 4.2E+05 | *0.23* | **2.4E+06** | 3.3E+05 | *0.21* |
| 15.13 | C11H9NO2 | [trans-3-Indoleacrylic acid](https://www.mzcloud.org/compound/reference/928) | **1.0E+07** | 2.4E+06 | **1.4E+07** | 8.8E+05 | *0.16* | **9.5E+06** | 8.3E+05 | *0.42* | **6.7E+06** | 8.6E+05 | *0.16* |
| 15.13 | C13H14N2O3 | [N-Acetyl-DL-tryptophan](https://www.mzcloud.org/compound/reference/814) | **3.2E+07** | 7.6E+06 | **4.5E+07** | 2.5E+06 | *0.14* | **3.0E+07** | 3.1E+06 | *0.40* | **2.1E+07** | 2.5E+06 | *0.15* |
| 15.21 | C15H16O6 | * [5-Hydroxy-7-(hydroxymethyl)-2-methyl-2-(5-oxotetrahydro-2-furanyl)-2,3-dihydro-4H-chromen-4-one](https://www.mzcloud.org/compound/reference/7593) | **2.8E+06** | 7.9E+05 | **8.5E+06** | 8.4E+05 | *0.01* | **6.4E+06** | 1.0E+06 | *0.04* | **4.1E+06** | 5.9E+05 | *0.17* |
| 15.62 | C17H25N5O | [2-[(3S)-1-(Cyclohexylmethyl)-3-pyrrolidinyl]-5-(1-methyl-1H-imidazol-5-yl)-1,3,4-oxadiazole](https://www.mzcloud.org/compound/reference/9965) | **7.9E+06** | 7.3E+05 | **8.7E+06** | 5.4E+05 | *0.27* | **9.5E+06** | 6.2E+05 | *0.12* | **9.5E+06** | 7.8E+05 | *0.15* |
| 15.62 | C9H8O4 | [Caffeic acid](https://www.mzcloud.org/compound/reference/337) | **3.5E+09** | 1.1E+09 | **7.4E+09** | 1.5E+09 | *0.08* | **5.8E+09** | 5.1E+08 | *0.11* | **6.5E+09** | 8.0E+08 | *0.07* |
| 15.71 | C11H10O4 | [Scoparone](https://www.mzcloud.org/compound/reference/7538) | **4.2E+07** | 1.4E+07 | **1.5E+07** | 9.9E+06 | *0.14* | **4.6E+07** | 2.1E+07 | *0.46* | **3.1E+07** | 2.3E+07 | *0.38* |
| 15.79 | C9H8O | * [trans-Cinnamaldehyde](https://www.mzcloud.org/compound/reference/1762) | **1.0E+07** | 4.4E+05 | **1.1E+07** | 1.4E+06 | *0.31* | **1.5E+07** | 2.7E+06 | *0.09* | **2.6E+07** | 2.0E+06 | *0.00* |
| 15.83 | C10H10O4 | * [Ferulic acid](https://www.mzcloud.org/compound/reference/421) | **3.0E+08** | 2.1E+07 | **3.2E+08** | 8.0E+06 | *0.25* | **4.0E+08** | 1.2E+07 | *0.02* | **4.2E+08** | 2.3E+07 | *0.02* |
| 15.85 | C30H46O5 | [(3β,5ξ,9ξ)-3,23-Dihydroxy-1-oxoolean-12-en-28-oic acid](https://www.mzcloud.org/compound/reference/7834) | **4.8E+07** | 5.0E+06 | **4.0E+07** | 2.2E+06 | *0.13* | **3.7E+07** | 1.5E+06 | *0.07* | **3.6E+07** | 5.3E+06 | *0.11* |
| 15.97 | C14H20O4 | [(8aR,12S,12aR)-12-Hydroxy-4-methyl-4,5,6,7,8,8a,12,12a-octahydro-2H-3-benzoxecine-2,9(1H)-dione](https://www.mzcloud.org/compound/reference/7607) | **3.5E+07** | 4.9E+06 | **2.9E+07** | 3.3E+06 | *0.25* | **3.1E+07** | 2.7E+06 | *0.30* | **2.5E+07** | 1.2E+06 | *0.10* |
| 15.98 | C13H18O | [Heptanophenone](https://www.mzcloud.org/compound/reference/1817) | **3.1E+07** | 3.1E+06 | **3.4E+07** | 4.1E+06 | *0.29* | **3.1E+07** | 3.7E+06 | *0.47* | **2.4E+07** | 1.9E+06 | *0.10* |
| 16.02 | C30H48O6 | [(1α,2α,3β,5ξ,9ξ,18ξ)-1,2,3,19-Tetrahydroxyurs-12-en-28-oic acid](https://www.mzcloud.org/compound/reference/7684) | **2.8E+07** | 6.3E+06 | **5.7E+07** | 1.4E+07 | *0.10* | **3.0E+07** | 2.9E+05 | *0.43* | **3.5E+07** | 6.2E+05 | *0.22* |
| 16.52 | C17H12O6 | * [4-(3,4-Dihydroxyphenyl)-6,7-dihydroxy-2-naphthoic acid](https://www.mzcloud.org/compound/reference/7610) | **1.2E+07** | 2.1E+06 | **3.0E+07** | 6.7E+06 | *0.05* | **3.3E+07** | 4.1E+06 | *0.01* | **1.9E+07** | 3.7E+06 | *0.10* |
| 16.53 | C10H10O3 | ** [4-Methoxycinnamic acid](https://www.mzcloud.org/compound/reference/3427) | **4.9E+07** | 3.4E+06 | **5.3E+07** | 1.2E+07 | *0.40* | **4.3E+07** | 2.8E+06 | *0.16* | **3.8E+07** | 2.3E+06 | *0.05* |
| 16.54 | C16H18O8 | [4-Methylumbelliferyl-α-D-glucopyranoside](https://www.mzcloud.org/compound/reference/241) | **3.0E+07** | 5.7E+06 | **3.1E+07** | 2.4E+06 | *0.46* | **3.6E+07** | 5.3E+06 | *0.30* | **3.1E+07** | 6.4E+06 | *0.47* |
| 16.63 | C20H32O2 | * [Arachidonic acid](https://www.mzcloud.org/compound/reference/2742) | **1.5E+07** | 7.0E+05 | **2.3E+07** | 4.8E+06 | *0.13* | **1.8E+07** | 2.9E+05 | *0.01* | **1.7E+07** | 3.9E+05 | *0.04* |
| 16.76 | C6H6O3 | [5-Hydroxymethyl-2-furaldehyde](https://www.mzcloud.org/compound/reference/630) | **6.3E+07** | 9.8E+06 | **5.9E+07** | 1.2E+07 | *0.42* | **4.3E+07** | 8.0E+06 | *0.13* | **4.4E+07** | 4.1E+06 | *0.11* |
| 16.80 | C9H8O3 | * [4-Coumaric acid](https://www.mzcloud.org/compound/reference/539) | **3.4E+07** | 9.5E+06 | **1.1E+08** | 2.7E+07 | *0.05* | **7.7E+07** | 4.2E+06 | *0.01* | **1.0E+08** | 1.2E+07 | *0.01* |
| 16.98 | C14H22O2 | [Isobornyl methacrylate](https://www.mzcloud.org/compound/reference/768) | **1.4E+06** | 1.8E+05 | **1.8E+06** | 6.0E+05 | *0.35* | **1.5E+06** | 4.9E+05 | *0.48* | **2.4E+06** | 6.3E+05 | *0.14* |
| 17.52 | C14H16O3 | [Sorbicillin](https://www.mzcloud.org/compound/reference/7826) | **9.9E+07** | 4.6E+06 | **1.1E+08** | 6.0E+06 | *0.16* | **1.1E+08** | 3.4E+06 | *0.19* | **8.7E+07** | 3.5E+06 | *0.08* |
| 17.52 | C8H8O | ** [Acetophenone](https://www.mzcloud.org/compound/reference/2744) | **1.1E+07** | 2.5E+05 | **1.2E+07** | 8.7E+05 | *0.19* | **1.2E+07** | 7.1E+05 | *0.24* | **9.6E+06** | 2.9E+05 | *0.02* |
| 17.52 | C15H14O4 | * [Yangonin](https://www.mzcloud.org/compound/reference/5539) | **1.2E+08** | 1.0E+07 | **1.9E+08** | 9.1E+05 | *0.00* | **1.9E+08** | 1.1E+07 | *0.01* | **1.3E+08** | 4.0E+06 | *0.23* |
| 17.90 | C12H14O4 | [Monobutyl phthalate](https://www.mzcloud.org/compound/reference/2821) | **7.5E+06** | 1.7E+05 | **7.6E+06** | 5.2E+05 | *0.41* | **8.3E+06** | 3.1E+05 | *0.06* | **7.1E+06** | 3.8E+05 | *0.24* |
| 18.22 | C14H14O4 | * [Columbianetin](https://www.mzcloud.org/compound/reference/7949) | **4.5E+06** | 8.7E+05 | **8.5E+06** | 4.3E+05 | *0.01* | **6.5E+06** | 7.9E+05 | *0.12* | **4.4E+06** | 1.8E+05 | *0.46* |
| 18.28 | C14H16O3 | [Dihydrokawain](https://www.mzcloud.org/compound/reference/7762) | **3.1E+07** | 2.5E+06 | **3.4E+07** | 3.1E+06 | *0.27* | **3.5E+07** | 1.5E+06 | *0.15* | **3.0E+07** | 1.3E+06 | *0.40* |
| 18.44 | C6H6O3 | [Pyrogallol](https://www.mzcloud.org/compound/reference/571) | **5.4E+06** | 1.9E+06 | **5.6E+06** | 9.7E+05 | *0.47* | **3.4E+06** | 5.6E+05 | *0.22* | **2.6E+06** | 1.4E+05 | *0.14* |
| 18.47 | C15H24O | * [(-)-Caryophyllene oxide](https://www.mzcloud.org/compound/reference/6554) | **5.5E+07** | 1.8E+07 | **1.8E+08** | 3.9E+07 | *0.04* | **3.8E+07** | 6.7E+06 | *0.26* | **2.1E+07** | 8.7E+05 | *0.10* |
| 18.51 | C18H28O3 | [12-Oxo phytodienoic acid](https://www.mzcloud.org/compound/reference/9879) | **5.7E+07** | 7.8E+06 | **7.2E+07** | 1.3E+07 | *0.23* | **6.3E+07** | 2.9E+06 | *0.29* | **6.3E+07** | 4.3E+06 | *0.32* |
| 18.71 | C24H34O8 | ** [Nandrolone glucuronide](https://www.mzcloud.org/compound/reference/7341) | **6.7E+07** | 1.5E+07 | **1.2E+08** | 2.0E+07 | *0.07* | **4.6E+07** | 5.6E+06 | *0.18* | **2.7E+07** | 7.3E+05 | *0.05* |
| 19.36 | C17H14O7 | [Aflatoxin G2](https://www.mzcloud.org/compound/reference/302) | **6.9E+06** | 1.3E+06 | **1.1E+07** | 3.0E+06 | *0.17* | **6.0E+06** | 5.2E+05 | *0.33* | **4.3E+06** | 1.4E+06 | *0.17* |
| 19.56 | C34H59NO15 | [Fumonisin B1](https://www.mzcloud.org/compound/reference/1220) | **1.3E+07** | 3.1E+06 | **1.8E+07** | 5.4E+05 | *0.15* | **1.4E+07** | 1.5E+06 | *0.43* | **1.3E+07** | 3.9E+05 | *0.48* |
| 19.58 | C12H24O3 | ** [Texanol](https://www.mzcloud.org/compound/reference/2649) | **1.3E+07** | 1.8E+05 | **1.2E+07** | 8.5E+05 | *0.18* | **1.2E+07** | 9.1E+05 | *0.20* | **1.1E+07** | 8.3E+05 | *0.05* |
| 19.59 | C16H12O6 | [Hispidulin](https://www.mzcloud.org/compound/reference/7411) | **1.0E+06** | 2.7E+05 | **3.2E+06** | 1.0E+06 | *0.09* | **1.7E+06** | 5.8E+05 | *0.22* | **9.5E+05** | 3.2E+05 | *0.45* |
| 19.65 | C20H28O2 | ** [Isotretinoin](https://www.mzcloud.org/compound/reference/1177) | **3.1E+06** | 2.3E+05 | **3.7E+06** | 1.7E+05 | *0.09* | **2.7E+06** | 1.1E+05 | *0.15* | **2.3E+06** | 1.1E+05 | *0.03* |
| 19.89 | C20H34O2 | [γ-Linolenic acid ethyl ester](https://www.mzcloud.org/compound/reference/6897) | **4.7E+06** | 1.0E+06 | **8.4E+06** | 1.6E+06 | *0.09* | **4.9E+06** | 7.2E+05 | *0.45* | **2.9E+06** | 1.7E+05 | *0.11* |
| 20.12 | C10H14O | [L-(-)-Carvone](https://www.mzcloud.org/compound/reference/1537) | **1.1E+07** | 1.8E+06 | **1.6E+07** | 8.9E+05 | *0.05* | **9.9E+06** | 1.3E+06 | *0.38* | **8.3E+06** | 2.2E+05 | *0.16* |
| 21.18 | C18H24O2 | [19-Norandrostenedione](https://www.mzcloud.org/compound/reference/1860) | **2.0E+07** | 2.9E+06 | **2.2E+07** | 7.0E+05 | *0.30* | **2.3E+07** | 1.7E+06 | *0.27* | **1.7E+07** | 7.4E+05 | *0.21* |
| 21.29 | C18H29NO4 | * [2-Methoxy-N-[(3S,3aS,8S,9S,9aS,9bS)-3,5a,9-trimethyl-2-oxododecahydronaphtho[1,2-b]furan-8-yl]acetamide](https://www.mzcloud.org/compound/reference/9640) | **5.9E+07** | 1.5E+07 | **5.7E+07** | 1.5E+07 | *0.47* | **7.8E+07** | 1.6E+07 | *0.26* | **1.4E+08** | 2.7E+07 | *0.05* |
| 21.36 | C17H14O6 | [Scrophulein](https://www.mzcloud.org/compound/reference/7753) | **1.6E+07** | 4.4E+06 | **4.2E+07** | 1.7E+07 | *0.15* | **1.4E+07** | 2.4E+06 | *0.40* | **1.6E+07** | 7.0E+06 | *0.48* |
| 21.39 | C10H8O3 | [4-Methylumbelliferone](https://www.mzcloud.org/compound/reference/240) | **1.3E+08** | 3.1E+07 | **1.2E+08** | 2.6E+07 | *0.45* | **1.4E+08** | 2.9E+07 | *0.44* | **1.6E+08** | 1.2E+07 | *0.25* |
| 21.53 | C21H32O4 | [(1S,4aR,5S)-5-[(3E)-5-Methoxy-3-methyl-5-oxo-3-penten-1-yl]-1,4a-dimethyl-6-methylenedecahydro-1-naphthalenecarboxylic acid](https://www.mzcloud.org/compound/reference/7868) | **2.7E+06** | 7.1E+05 | **4.4E+06** | 1.2E+06 | *0.20* | **1.9E+06** | 2.1E+05 | *0.21* | **1.2E+06** | 1.2E+04 | *0.08* |
| 21.74 | C20H20O4 | [Calocarpin](https://www.mzcloud.org/compound/reference/7825) | **3.1E+06** | 8.8E+05 | **5.2E+06** | 9.7E+05 | *0.12* | **2.1E+06** | 6.8E+05 | *0.26* | **2.2E+06** | 2.6E+05 | *0.24* |
| 22.18 | C17H12O6 | ** [Aflatoxin B1](https://www.mzcloud.org/compound/reference/300) | **8.0E+06** | 6.9E+05 | **6.4E+06** | 1.0E+06 | *0.18* | **2.6E+06** | 4.6E+05 | *0.00* | **1.5E+06** | 3.5E+05 | *0.00* |
| 22.35 | C13H24N2O | * [N,N'-Dicyclohexylurea](https://www.mzcloud.org/compound/reference/2978) | **4.3E+06** | 2.6E+04 | **5.6E+06** | 5.4E+05 | *0.06* | **5.2E+06** | 4.9E+05 | *0.10* | **5.0E+06** | 2.2E+05 | *0.04* |
| 22.66 | C16H24O3 | ** [Methyl 3,5-di-tert-butyl-4-hydroxybenzoate](https://www.mzcloud.org/compound/reference/804) | **6.2E+06** | 1.0E+06 | **8.2E+06** | 1.0E+06 | *0.16* | **4.5E+06** | 3.9E+05 | *0.14* | **2.1E+06** | 4.6E+05 | *0.02* |
| 22.66 | C16H12O5 | [Glycitein](https://www.mzcloud.org/compound/reference/428) | **1.6E+07** | 4.8E+06 | **4.1E+07** | 1.2E+07 | *0.10* | **2.2E+07** | 4.7E+06 | *0.28* | **2.0E+07** | 7.1E+06 | *0.36* |
| 22.66 | C19H18O8 | [5,2'-Dihydroxy-6,7,8,6'-tetramethoxyflavone](https://www.mzcloud.org/compound/reference/7780) | **4.2E+07** | 1.0E+07 | **6.1E+07** | 1.4E+07 | *0.22* | **4.1E+07** | 6.4E+06 | *0.47* | **3.1E+07** | 1.0E+07 | *0.27* |
| 22.92 | C18H39NO3 | * [2-Amino-1,3,4-octadecanetriol](https://www.mzcloud.org/compound/reference/545) | **2.9E+07** | 1.1E+07 | **2.4E+07** | 4.8E+05 | *0.35* | **2.7E+07** | 2.4E+06 | *0.43* | **6.2E+07** | 2.6E+06 | *0.04* |
| 22.95 | C10H16O | [(-)-Camphor](https://www.mzcloud.org/compound/reference/2759) | **5.6E+07** | 2.3E+07 | **1.1E+08** | 4.0E+07 | *0.21* | **1.9E+07** | 5.1E+06 | *0.14* | **5.8E+07** | 1.9E+07 | *0.48* |
| 23.02 | C30H44O3 | [Boldenone undecylenate](https://www.mzcloud.org/compound/reference/7234) | **1.2E+07** | 2.4E+06 | **2.4E+07** | 6.2E+06 | *0.11* | **1.1E+07** | 6.3E+05 | *0.46* | **1.4E+07** | 1.1E+06 | *0.27* |
| 23.06 | C18H26O2 | [19-Nortestosterone](https://www.mzcloud.org/compound/reference/288) | **5.2E+06** | 1.5E+06 | **6.6E+06** | 2.2E+06 | *0.34* | **3.4E+06** | 1.2E+05 | *0.18* | **2.5E+06** | 2.4E+05 | *0.11* |
| 23.14 | C17H14O6 | [Aflatoxin B2](https://www.mzcloud.org/compound/reference/6148) | **2.4E+06** | 4.6E+05 | **5.0E+06** | 1.3E+06 | *0.10* | **2.5E+06** | 4.9E+05 | *0.47* | **2.9E+06** | 8.5E+05 | *0.35* |
| 23.52 | C20H28O3 | [(1S,4aS,5R)-5-[2-(3-Furyl)ethyl]-1,4a-dimethyl-6-methylenedecahydro-1-naphthalenecarboxylic acid](https://www.mzcloud.org/compound/reference/7848) | **1.6E+07** | 2.0E+06 | **1.6E+07** | 1.4E+06 | *0.49* | **1.7E+07** | 8.7E+05 | *0.40* | **1.3E+07** | 4.9E+05 | *0.16* |
| 24.17 | C30H48O5 | [(3β,5ξ,9ξ)-3,6,19-Trihydroxyurs-12-en-28-oic acid](https://www.mzcloud.org/compound/reference/7810) | **1.1E+08** | 1.6E+07 | **2.3E+08** | 7.7E+07 | *0.14* | **1.1E+08** | 1.5E+07 | *0.48* | **1.3E+08** | 1.8E+07 | *0.23* |
| 24.18 | C30H46O4 | [18-β-Glycyrrhetinic acid](https://www.mzcloud.org/compound/reference/1281) | **1.1E+08** | 1.4E+07 | **1.9E+08** | 5.4E+07 | *0.17* | **9.8E+07** | 1.5E+07 | *0.31* | **1.3E+08** | 1.6E+07 | *0.25* |
| 24.27 | C8H7N | [Indole](https://www.mzcloud.org/compound/reference/444) | **5.2E+07** | 3.2E+06 | **6.1E+07** | 8.4E+06 | *0.23* | **5.0E+07** | 4.7E+06 | *0.37* | **4.1E+07** | 3.0E+06 | *0.06* |
| 24.96 | C18H30O2 | [α-Linolenic acid](https://www.mzcloud.org/compound/reference/6865) | **6.1E+06** | 4.7E+05 | **8.8E+06** | 2.4E+06 | *0.21* | **5.6E+06** | 6.5E+05 | *0.32* | **5.1E+06** | 2.5E+05 | *0.10* |
| 25.95 | C23H22O8 | [5,6a-Dihydroxy-2-isopropenyl-8,9-dimethoxy-1,2,12,12a-tetrahydrochromeno[3,4-b]furo[2,3-H]chromen-6(6aH)-one](https://www.mzcloud.org/compound/reference/7877) | **1.6E+06** | 3.3E+05 | **9.4E+06** | 4.5E+06 | *0.11* | **2.5E+06** | 5.4E+05 | *0.15* | **3.3E+06** | 1.0E+06 | *0.14* |
| 26.37 | C18H30O3 | [13(S)-HOTrE](https://www.mzcloud.org/compound/reference/9864) | **1.9E+07** | 5.8E+06 | **3.0E+07** | 8.0E+06 | *0.22* | **1.4E+07** | 2.1E+06 | *0.25* | **8.8E+06** | 1.1E+06 | *0.11* |
| 26.40 | C19H18O3 | * [Tanshinone IIA](https://www.mzcloud.org/compound/reference/5848) | **1.1E+07** | 1.1E+06 | **1.3E+07** | 2.0E+06 | *0.23* | **1.3E+07** | 2.2E+06 | *0.22* | **1.9E+07** | 2.2E+06 | *0.03* |
| 26.41 | C16H21NO3 | * [MDPV](https://www.mzcloud.org/compound/reference/2263) | **3.6E+06** | 5.2E+05 | **6.3E+06** | 8.8E+05 | *0.05* | **8.3E+06** | 1.9E+06 | *0.06* | **8.5E+06** | 2.3E+06 | *0.08* |
| 28.71 | C16H22O4 | [Dibutyl phthalate](https://www.mzcloud.org/compound/reference/32) | **1.2E+07** | 3.6E+06 | **1.8E+07** | 2.5E+06 | *0.16* | **1.9E+07** | 1.0E+06 | *0.10* | **1.5E+07** | 4.9E+06 | *0.35* |
| 29.55 | C22H36O3 | * [3-Methyl-5-(5,5,8a-trimethyl-2-methylene-7-oxodecahydro-1-naphthalenyl)pentyl acetate](https://www.mzcloud.org/compound/reference/7393) | **1.4E+06** | 3.2E+05 | **3.6E+06** | 1.6E+06 | *0.16* | **2.5E+06** | 3.3E+05 | *0.06* | **3.6E+06** | 7.4E+05 | *0.04* |
| 29.61 | C9H14 | [1,2,3,4-Tetramethyl-1,3-cyclopentadiene](https://www.mzcloud.org/compound/reference/3247) | **6.1E+06** | 2.2E+05 | **8.8E+06** | 1.4E+06 | *0.10* | **6.7E+06** | 7.0E+05 | *0.28* | **5.7E+06** | 4.4E+05 | *0.25* |

* upregulated compound. ** downregulated compound.
