# Supplementary material for: Increase of secondary metabolites in sweet basil (Ocimum basilicum L.) leaves by exposure to N2O5 with plasma technology
Source: Sci Rep. 2024 Jun 4;14:12759. doi: 10.1038/s41598-024-63508-8 (PMC11150270; doi:10.1038/s41598-024-63508-8)
Supplement: Supplementary file 2 — Supplementary Table 2. [file 41598_2024_63508_MOESM2_ESM.docx]

| Supplementary Table 2. Primers and amplicon length of the genes that were analyzed in this study | | | | |
| --- | --- | --- | --- | --- |
| Gene | Primer Name | Sequence (5’ → 3’) | Product size (bp) | Reference |
| *C4H* | C4H_F | GCCAACAACCCCGCTCAATG | 119 | Abdollahi et al., 2017 |
|  | C4H_R | CCAACGCCGAAGGGGAGGTATC |  |  |
| *4CL* | 4CL_F | TCGTAGACAGGGTGAAGGAGC | 138 | Abdollahi et al., 2017 |
|  | 4CL_R | CTTCACCAGCAGCCTCATCT |  |  |
| *CCR* | CCR2_350F | GCGTGGTGTTAACCTCCTCA | 140 | This study^a^ |
|  | CCR2_489R | CACAGCCTTCCCATAGCAGT |  |  |
| *CAD* | CAD_F | GCGGATTTGCTGGGGCTATG | 87 | Abdollahi et al., 2017 |
|  | CAD_R | GTAGTGGTGCTGCCTGTTCC |  |  |
| *CAAT* | ObCAAT1_q_F | AAGATGAGAAGAAATTAGTT | 172 | Dhar et al., 2020 |
|  | ObCAAT1_q_R | TCAAAATCGTGGCCTCCTTTGT |  |  |
| *EGS* | q_EGS1-F | GGTGCCATCATAGTCAAGGG | 205 | Reddy et al., 2021 |
|  | q_EGS1-R | GCAATGCGTTTATTCTGTCCT |  |  |
| *EOMT* | EOMT433F | ACCCAGTTTGAGGCAGCAAA | 106 | This study^b^ |
|  | EOMT538R | CAAGCCTTGCGTCACAACTC |  |  |
| *CIN* | CIN619F | CTGAATGAGGGCCACCATGA | 118 | This study^a^ |
|  | CIN736R | ATGCATCAATGCACCAGCTT |  |  |
| *LIS* | LIS1117F | GAAGCCCTCTTCTTCACCCC | 101 | This study^a^ |
|  | LIS1217R | CCCTTCCACGTTCGATTCCA |  |  |
| *OPR3* | OPR3.1869Fr | GGCACTGCAGACGAGGAGGC | 141 | This study^c^ |
|  | OPR3.2009R | TCCATACGCCACCAAGTCAG |  |  |
| *Tubulin* | TUB2078F | AGTTCACGGAGGCAGAGAGC | 122 | This study^c^ |
|  | TUB2190R | CTTCCTCCACACCATCCTCC |  |  |
| ^a^The primers were designed with cDNA sequences that were determined by RNA sequencing analysis as shown below.  ^b^The primers for *EOMT* gene that were described in Abdollahi et al. (2017) could not produce single PCR product in our PCR conditions. Therefore, we designed primers with AF435008, XLOC_068107 and XLOC_068808 that seems to encode EOMT (Gonda et al., 2020).  ^c^Genomic DNA containing *OPR3* gene (Chr: scaffold10, 7268155 - 7270534) and that containing *beta-tubulin* gene (Chr: scaffold4172, 15792757 - 15795312)) in *Ocimum basilicum* cv. Perrie (Unit of Aromatic and Medicinal Plants scaffolds v0.1) were identified by Blast research with Arabidospsis *OPR3* gene (AT2G06050) and *Ocimum basilicum beta-tubulin* gene (MH620961.1), respectively, at https://genomevolution.org/coge/, and then the primers of the *beta-tubulin* gene were designed. For *OPR3* gene, genomic DNA was used to identify the cDNA that was predicted by RNA-Seq, and then the primers of *OPR3* gene. | | | | |

**cDNA sequences determined in this study**

*>ObOPR3* cDNA

ATTTTATTTATTAGCATTTGACAGTACCAAATACGAAACAGCAGCCACAGTTTGACTAAAAAAGAATAGCCCGAAAACTAGTGAAATGATTTACATATGAAAATCGTGGACACATAAACTCATTTATTTTGCCAAACTCTTCAACGTTCCCCACTATTTTCTACACAACACAAGCATTCATTTTTCTGCCACCGGCCACAACCGCAGCTCTTCCTCCTCTACTCCGCCGTCCAAACGAACACCATTAAATACTATCTAGCTTCCTGAAGGAACACAGAGCTACGCAGCAATGGCAGAAACGGCGTCGTCTCTCTTCTCCCCTTACAAGATGGGCAAATTTCATCTCTCGCACAGGGTGGTTCTGGCTCCGATGACTCGATGCAGGGCGTTAAATACCATGCCGAATTCTGCACTGGCGGAGTACTACGCTCAGAGAGCTACGGAAGGAGGATTTCTCATCACGGAGGGCACCATGATCTCCCCCTATGCTGCGGGGTTTCCGCACGTGCCAGGCATCTTCAACCAGGAGCAAGTGGAGGCTTGGAAGGAGGTAGTTGAGAGGGTGCACGCTAAGGGCGCTGTTATATTCTGTCAGCTCTGGCACGTCGGCCGCGCTTCTCATCCAGGTTGATTTAATATTCATGACTAACGCCGATCATGTGCCGTTTAGATAGTTTCGCCTACATCTATCTACATATTGGGTTTTCTTACATGGATATATTTAACTATAAATTCTTGTTGGCCTTTTTATTAGAAATTCTTGGTGTTAGACATGAGAGAATTTGACCTCTAGGTTCAATTAATATCTACCATTATATGGTTTTAGTCAATATTTATGCTTTTTGCTTAGTTAATTATGCTTGAAAAAGCAAATAGAGAAGCATGATGACTTAGTATGATTTATATGTACAGAGGTGGTGAATCATACAAGAGGAAAAAAAAAAAAAAATAGTATACAGGAAATAGGATAATTTTGAACCACGAGTCAATAAAATCTATTGTTGGGTTTTTTCATTTTTACTTGTTTATTTGTTAAATTCAGAAATCATTTACAAAACATAGATTCATCAAGATTAAAAATGAAAATATATCAACCATATTATTTAGCTAAAAATTTCTTGTTTTTCTTATAGAATCCAAAATACTATGTATATATAACCATGTTTTGATATATATGTAGAAAACAAATAGTGTTGAACTACAAATCATGTTTCAACATTTCACATTGAAGAGGAAATTCTATGTTACATACTTTGGCACTATCAAACGCCAAAGTCGGCACCTAGGAGTTTCCCAACATGACATTCCTTTCTGTTTACCTATTGAATTGGGCATAGCATGCTCTATAGAAATGCAGCATTACTTGATCCAAAGTCAGCAAGTTTTGAATCCAGACTTTTCTTGAATTATGGCATATTTGTGAATGGAACCCATTTCCCAATAAAATGGAGAGTGAATGGAAACCATTTCCCAATTTAGTAATTGTTGTTTCAAAAATGAAAATGTATGTTGGCACAAACAGAACTTCAGCCTGGTGGTGAGGCACCAATATCCTCGACGGACAAGCCGATATCCAAGAGATGGAGAGTGCTGCTTCCAGATGGTAAATATGCGGTTTATCCAAAGCCCCGCAAATTGGAGACTGATGAGGTGCCTCAGATTGTTCAACAGTATCGTCAAGCCGCCATTAATGCCATTCTAGCAGGATTTGATGGCATTGAGATCCATGGAGCTCATGGTTACCTCCTTGATCAATTCATGAAGGATGGGATCAACGAACGGACAGATGAGTACGGTGGATCCCTCCAAAATCGCTGCAAATTCATCATCCAAGTAGTTGAAGCTGTTGTTTCTGCAATTGGTGCTGATCGAGTAGGTGTCAGAATCTCACCTGCAATTGATCATCTTGATGCGATGGACTCTGATCCACTCAACCTTGGACTAGCTGTGATTGAGAGACTCAATAAACTACAGTTAGCAAGCGGTTCAAAGCTCGCTTATCTGCACGTGACTCAGCCACGATACACAGCCTACGGGCAAACAGAATCGGGCAGGCATGGCACTGCAGACGAGGAGGCACAGATGATGAGGACTTGGCGAAGGACGTATGAGGGAACATTCATTAGTAGTGGTGGATTCACTAGACAGCTAGGGATTGAAGCAGTGGCACAAGGGGATGCTGACTTGGTGGCGTATGGACGGCTCTTTATTTCGAACCCAGATTTAGTTCTCAGGCTCAAGCTGAACGCGCCTTTAACCAAGTATGTGCGAGCTACATTTTACACGCATGACCCTGTTGTGGGATATACTGATTATCCTTTTCTGAAATCTGATGGTGAAAAACCTGCATCACGACTTTAAGACGTAGTACTGGTACCTTTGGAGGCTACAGCTTGTTTAAGAATGTGTATTTTTCTGTTG

*CCR* (*cinnamoyl-CoA reductase*) cDNA

ATGCCATCGGTTCATGGGAAAGTCGTCTGCGTCACCGGCGCCGGAGGCTTCATTGCTTCCTGGCTGGTTAAATTGCTCCTCGAGAAAGGCTATACCGTCAGAGGAACCGTCAGAAACCCTGATGATCCGAAGAATTCGCATTTGCGAGAGCTTGAAGGAGCCGCTGAGAGGCTGATTCTGTGCAGAGCTGATCTTAACGTTTATGAGAGTCTGCGCGAAGCCATTAATGGCTGCGATGGCGTCTTCCACACGGCGTCGCCGGTCACCGATGATCCAGAACAAATGGTGGAGCCGGCGGTAGAGGGCGCGAAGAGCGTGATACGTGCGGCGGCGGAGGCCAAAGTTCGGCGCGTGGTGTTAACCTCCTCAATTGGTGCAGTGTACATGGACCCCAACAGGGATCCTGATAAAGTTGTGGATGAGACTTGTTGGAGTGATCTTGAATTCTGCAAAAACACGAAGAACTGGTACTGCTATGGGAAGGCTGTGGCGGAGCAAGCTGCATGGGAAACAGCTAAGGAATTAGGGGTGGATTTGGTGGTGCTGAACCCGGTTTTGGTGCTTGGCTCATTGCTCCAGCAAACTGTGAATGCCAGTGTGCTTCACATACTCAAATATTTAACTGGCTCTGCAAAAACTTATGCCAATTCTATTCAGGCATATGTCGATGTGAAAGATGTCGCCTTGGCCCACATCCTGTTGTTCGAGAATCCAGCGGCATCTGGCCGGTATCTCTGCGCGGAGAGCGTCCTCCACCGCGGCGAGGTGGTGGAGATTCTTGCCAAGTTCTTCCCGGAGTATCCTATACCTACCAAGTGTTCAGATGAGAAAAACCCAAGGAAAAAACCATATAAATTTTCAAACCAAAAGCTGAAGGATTTGGGGCTAGAATTCACCCCAGTGAGGCAAAGTTTATACGACACAGTGAAAAGTCTTCAAGAAAAAGGGCATCTTCCGATCCCAACTCAGAACGAGGACCCTGTTCGTATTCATCCTTAG

*CIN* (*1,8-Cineole synthase*) cDNA

ATGTCTTCAAAACATTATGCTAATAATCCTTGCAGTAGCATTCCTGCAGCTGCTGCTCGCCTCCGGCTTCGTTGTTTGTCGCAAGAACTAACTGATCACGTCCCAGCACGAGGACGACGAACGGGGAACTACAGTCCCTCCCTTTGGGAAACCGATTATATTCAATCGCTCCAAACTGACTACAAGGAAGAGAAGCATGTGAGGAGGATTTGTGGGCTAATTAAGCAAGTAAAGACGCTATTGGAGAAAGAAAAGTGCCTCGTTCCACAGCTAGAATTGATTGATGATCTTCAAAGGCTAGGTCTCTCTGACCATTTCCAAAATGAAATCAAACAAATGTTAACCTCTATACACCTGCATTACACCAACAACCAGACGACACAGGAAGATGACTTGTACTCCACAGCTCTTACATTCAGACTCCTTAGACAACACGGCTTTCAAGTTTCTCAAGAGGTGTTTGACTGCTTCACAAATGAAAAGGGTGAATTCATGCCAAGTCTTAGCAGCGACACTAGAGGGATATTGCAATTGTATGAAGCATCTTTCTTATTGACACGAGGCGAACACACTCTTGAAATAGCAAAAGAATTTGCAACCAACATTCTACTGAAATTTCTGAATGAGGGCCACCATGAAATTGATGGTAATATTTTATTTTTGGTACGTAATTGTTTGGACATCCCAATTCATTGGAGGATTCAACGACCAAATATAAGCTGGTGCATTGATGCATACGAGCAGAGACTTGACATGAATCCAGTTGTGTTGGAGCTTGCCAAACTAGACTTCAATGTTGTGCAAGCACAATATCAAGAAGAACTACAAGAATGCTTAAGATGGTGGAAAAACACATGCCTTGTTGAAAAGCTCCCATTTGTTAGAGATAGAGTTGTGGAAAGCTATTTTTGGACTATCGGCATCATTGGGCCTCGTCAGTATAGAAAGGGAAGATTAATGGTAAACAAAGTTATAGCCCTATCAACAGTGATGGATGATATTTATGATGTCTATGGAACATTAGAAGAACTAGAACTTTTCACAGAGGCAATTCAAAGATGGGATTTCAGTTCAATCGACCAACTTCCTTATTACATGCAACTATGTTATCTCGCAATTGTGAATTTCGTCGACGACACATCATATGATGTTTTGAAGGAGAAAGGTTTCAACATCATTCCATATCTTCAAAAATCGTGGAGGGATTTGGCGGAGGCATATATGGTGGAGGCGAAATGGTACAACAGCGGATCAAAACCAAAACTGGAAGAGTACGTGAATAACGCGTGGGTTTCAATCGCTGGCCCTGCAATATTATCACATGCTTTTTTGGGTGTAGCAGATGCAGATTTGGTATCCAAGGACAGTGTTGAGGATTTCTATAATAACAATATACTTCGCTCCTCATCCATTATTTTACGCCTTGCTGATGATTTAGCTACCTCCCAGAATGAGATGGAAAGAGGCGACGTGCCAAAATCGATTCAGTGCTACATGAACGACAATAATGCTTCGGAGGAAGAGGCACGAGAGCATGTGAGAAGGATGATTGCAGAGACGTGGAAAAAGATGAATGAGGAAAGGGTGTCATGTGTGTATTCTACATTGATGGAATGTGCTGTGGGTATGGCTAGAATGGGGCAATATATGTACCATTATGGAGATGGCCATGGCATACAACCCCCTCAAACACATCTACAAATGTCAAAATTGTTGTTCCATCCTCTTACTTGA

*LIS* (*Linalool synthase*) cDNA

ATGGAGTTTGCGGAGGCTCGCCTGAGACGATCACTGTCGGAGTTGGCGGCGCCGCTTCGTGGTGAGGTGGCGCAAGCCCTAGATGTGCCGAGGCATCTGAGAATGGCGAGGTTGGAAGCGAGGCTATTCATCGAGCAGTATGGCAAACAGAGCGATCATGATGGAGAGCTTTTGGAGCTAGCAATTTTGGATTATAATCAAGTTCAGGCTCAACACCAATCCGAACTCACTGAAATTACCAGGTGGTGGAAGCAACTGGGTTTGGTGGAAAAGTTGGGTTTCGGTAGAGACAGAGCGTTGGAGTGCTTTATGTGGACCATGGGGATCCTACCTCACCCTAAATACTCTTCTTCTAGAATAGAATCAGCCAAGGCAGCTGCTCTTCTGTACGTCATCGATGATATTTTCGATACCTATGGCAAAATGGACGAACTCATCCTCTTCACCGACGCAATTCGAAGATGGGATTTGGAAGCAATGGAGGGTCTACCCGAGTACATGAAAATATGCTACATGGCGTTGTACAACACCACCAACGAAATATGCTACAAAGTTCTCAAGGACACTGGACGGATCGCCCTCCCTAACCTCAAATCTACGTGGATAGATATGATTGAAGGTTTCATGGTGGAAGCAAAATGGTTCAATGGTGGAAGTGCACCAAAGTTGGAAGAATACATTGAGAATGGGGCATCAACTGTAGGGGCATACATGGTTCTTGTCCACCTATTCTTTCTCATTGGAGAAGGTCTCACCCACCAAAATGTCCTATTTTTCAAACAAAAACCCTATCACAAGCTTTTCTCCGCCGCCGGCCGGATTTTTCGCCTTTGGGACGATCTTGGAACTTCTCAGGAGGAGGAAGAACGAGGAGATATGGCGTCAAGTATACGGTTATTTATGAAAGAGTACAAGTTGTCGACGGTAGAGGAGGCTAGAAGTTGCGTTTTGGAAGAGATATCCCGTTTATGGAAGGATCTTAATGAAGGGCTCATCAGTATAAAGGACGTCTTGCCATTAACCATAGTCAAAGTCGCACTTAACATTGCACGAACTTCTCAAGTTGTATACAAGCACGAACAACATACATATATGTTGAGTGTTGATAATTACGTGGAAGCCCTCTTCTTCACCCCTCTTCTTTCTTCTTAGCTCATTTTTTTTTTAAACAAAGAAAGAAATCTAGCTAAACCAGACTGGAATCGAACGTGGAAGGGCAACTCCAACCAAGTCGTAGTTCAACCACGTGTTCCGTCTTGTTCTTTCTTCTTAGTTCATCTTCTTTAATATGTATGTACTCGAGAATAATGCCCAATAAATTCGTGAAAATCATGAATACATTAATTATCATGACGACTCACGAGACTTTGCTTAAAA

**References**

Abdollahi Mandoulakani B, Eyvazpour E, Ghadimzadeh M. The effect of drought stress on the expression of key genes involved in the biosynthesis of phenylpropanoids and essential oil components in basil (*Ocimum basilicum* L.). Phytochemistry. 2017 Jul;139:1-7. doi: 10.1016/j.phytochem.2017.03.006.

Dhar N, Sarangapani S, Reddy VA, Kumar N, Panicker D, Jin J, Chua NH, Sarojam R. Characterization of a sweet basil acyltransferase involved in eugenol biosynthesis. J Exp Bot. 2020 Jun 22;71(12):3638-3652. doi: 10.1093/jxb/eraa142.

Reddy VA, Li C, Nadimuthu K, Tjhang JG, Jang IC, Rajani S. Sweet Basil Has Distinct Synthases for Eugenol Biosynthesis in Glandular Trichomes and Roots with Different Regulatory Mechanisms. Int J Mol Sci. 2021 Jan 12;22(2):681. doi: 10.3390/ijms22020681.

Gonda I, Faigenboim A, Adler C, Milavski R, Karp MJ, Shachter A, Ronen G, Baruch K, Chaimovitsh D, Dudai N. The genome sequence of tetraploid sweet basil, Ocimum basilicum L., provides tools for advanced genome editing and molecular breeding. DNA Res. 2020 Dec 3;27(5):dsaa027. doi: 10.1093/dnares/dsaa027.
